# The use of three-dimensional primary human myospheres to explore skeletal muscle effects of in vivo krill oil supplementation

**DOI:** 10.1007/s44164-025-00087-6

**Published:** 2025-04-30

**Authors:** Andrea Dalmao-Fernandez, Parmeshwar B. Katare, Hege G. Bakke, Håvard Hamarsland, Stian Ellefsen, Sachin Singh, Tuula Anneli Nyman, Eili Tranheim Kase, Arild C. Rustan, G. Hege Thoresen

**Affiliations:** 1 Section for Pharmacology and Pharmaceutical Biosciences, Department of Pharmacy, University of Oslo, Oslo, Norway; 2 Faculty of Social and Health Sciences, Section for Health and Exercise Physiology, Inland Norway University of Applied Sciences, Lillehammer, Norway; 3 Innlandet Hospital Trust, Lillehammer, Norway; 4 Department of Immunology, Institute of Clinical Medicine, University of Oslo and Oslo University Hospital, Oslo, Norway; 5 Department of Pharmacology, Institute of Clinical Medicine, University of Oslo, Oslo, Norway

**Keywords:** Skeletal muscle cells, 3D cell cultures, Krill oil, Energy metabolism, Motor proteins

## Abstract

**Purpose:**

Supplementation with krill oil has shown effects on whole-body lipid and glucose metabolism, as well as on skeletal muscle strength and function. We previously showed that krill oil intervention in vivo promoted fatty acid metabolism and protein synthesis in cultured human myotubes in a two-dimensional (2D) model. The aim of this study was to explore the effects of krill oil supplementation in vivo in a 3D myosphere model, and to compare a the human skeletal muscle 3D cell model to a 2D model.

**Methods:**

Myospheres were formed from myoblasts obtained before and after 7 weeks of in vivo krill oil intervention. Glucose and oleic acid metabolism were assessed, and transcriptomic and proteomic analyses were performed.

**Results:**

In vivo intervention with krill oil increased glucose metabolism in myospheres, while no effect was observed on fatty acid metabolism. Transcriptomic analyses of myospheres after krill oil intervention showed increased expression of genes involved in pathways like motor proteins and hypertrophy, as well as in calcium signaling, of which motor proteins and hypertrophy pathways have not been described in 2D myotube cultures. Proteomic analyses after krill oil intervention showed increased expression of proteins in glycolysis/gluconeogenesis and fatty acid degradation. Comparison of proteins expressed in the 3D myosphere model and the 2D myotube model at the basal level showed that in myospheres, mitochondrial gene expression and translation dominated, while in 2D cultures, mitochondrial organization and response to oxidative stress were more important.

**Conclusion:**

These findings suggest that in vivo krill oil intervention induces different metabolic effects when comparing 3D and 2D cultures. In contrast to the 2D model, data obtained with the 3D model showed gene expression changes that are more compatible with previously observed results in vivo concerning skeletal muscle motoric function. Hence, the 3D cell model might better reflect krill oil-induced modifications in skeletal muscle performance in vivo than the 2D model.

**Supplementary Information:**

The online version contains supplementary material available at 10.1007/s44164-025-00087-6.

## Introduction

Dietary supplementation of krill oil, containing omega-3 fatty acids, palmitoleic acid and the antioxidant astaxanthin, has been shown to have several beneficial effects on human health (reviewed in [1, 2]). In human studies, the effects of krill oil on lipid metabolism have primarily been documented, including lowering of triacylglycerol, total cholesterol and low-density lipoprotein (LDL)-cholesterol [1, 2]. In animal studies, improvement of glucose metabolism after krill oil has also been shown (see e.g. [3–5]), as krill oil feeding of mice on high-fat diet reduced blood glucose [3–5] and improved glucose tolerance [3] and insulin sensitivity [4]. In skeletal muscle, krill oil supplementation has been shown to increase muscle strength and power [6], as well as recovery of injury caused by resistance exercise [7].

We have previously shown that in vivo supplementation with krill oil for 7 weeks induced fatty acid oxidation and leucine accumulation in cultures of skeletal muscle cells harvested from biopsies obtained after the intervention, compared to cells from the same individuals obtained before the intervention [8]. The myotube cultures used were two-dimensional (2D) cultures, the most widely used model for culturing skeletal muscle cells. Myotube 2D cultures are widely used to study e.g. proliferation, differentiation and energy metabolism, but several parameters measured in 2D muscle cell models are not comparable to those found in vivo in muscle biopsies, such as fiber type, mRNA expression, oxidative energy metabolism and insulin response [9]. Due to these limitations, there is growing interest in establishing three-dimensional (3D) muscle cell models. The 3D cell culture models are claimed to be more representative for skeletal muscle in vivo and the use of these models might thereby provide more physiologically relevant data (reviewed in e.g. [10–13]). Different 3D models of skeletal muscle have been described, including myosphere, myobundle and bioengineered muscle constructs, using both primary muscle-derived cells [10–12] and embryonic or induced pluripotent stem cells [13–15]. Recently, we have described the development of a 3D primary myosphere model from human satellite cells without external matrix support as an easy and low-cost tool to study molecular mechanisms of energy metabolism [16]. While 2D myotube cultures are largely glycolytic [9], myospheres formed from human primary myoblasts showed increased lipid oxidative metabolism compared to the 2D myotube model [16].

The aim of this study was to examine the 3D myosphere model by exploring the effects of in vivo krill oil supplementation on energy metabolism and transcriptomic and proteomic changes in the myosphere model in comparison with previous data obtained from a 2D myotube model [8]. To further study the differences between the 3D and 2D cell models, the expression of proteins before the intervention (e.g. at the basal level) of the two cell models were compared.

## Materials and methods

### Materials

Dulbecco’s Modified Eagle’s Medium (DMEM)-Glutamax™ low glucose, Dulbecco’s Phosphate Buffered Saline (DPBS; without Ca^2+^ and Mg^2+^), penicillin–streptomycin (10000 IE/ml), amphotericin B, human epidermal growth factor (hEGF), trypsin–EDTA, fetal bovine serum (FBS), Paraformaldehyde (PFA), TRIzol™ Reagent, Power SYBR® Green PCR Master Mix, High-Capacity cDNA Reverse Transcription Kit, MicroAmp® Optical 96-well Reaction Plate, MicroAmp® Optical Adhesive Film, primers for PCR, Nunclon™ Sphera™ 96 Well Round (U) Bottom Plate Low-Attachment Surface, and Nunc™ Cell and Culture Treated Flasks with Filter Caps were from Thermo Fisher Scientific (Waltham, MA, US). D-[^14^C(U)]glucose (3.0 mCi/mmol) and [1-^14^C]oleic acid (OA, 59.0 mCi/mmol) were from PerkinElmer NEN® (Boston, MA, US). Ultima Gold™ XR, UniFilter®−96 GF/B microplates, 96-well Isoplate®, and TopSeal®-A transparent film were obtained from PerkinElmer (Shelton, CT, US). 4-(2-hydroxyethyl)−1-piperazineethanesulfonic acid (HEPES), bovine serum albumin (BSA), dexamethasone, gentamicin, L-glutamine, L-carnitine, trypan blue 0.4% solution, D-glucose, oleic acid (OA, 18:1, n- 9), Chloroform, Isopropanol and Triton-X100 were purchased from Sigma-Aldrich (St. Louis, MO, US). Sodium dodecyl sulfate (SDS) and Bio-Rad protein assay were purchased from Bio-Rad (Hercules, CA, US).

### Clinical trial and donor characteristics

The study is part of a larger trial, registered at ClinicalTrial*.*gov (ID: NCT04279951). In the current cohort, untrained, non-smoking human participants received a daily krill oil supplement (1 g/day, equivalent to four servings of fatty fish per week [17]) for 7 weeks, containing 0.12 g EPA, 0.065 g DHA, in total 0.24 g omega-3 polyunsaturated fatty acids, 0.03 g palmitoleic acid (16:1, omega-7), > 42% phospholipids, and > 0.2 mg of astaxanthin. Krill oil was provided by Rimfrost AS (Ålesund, Norway). Skeletal muscle biopsies were obtained from the *musculus vastus lateralis* on both the first and last days of the study, and these muscle biopsies underwent processing to isolate satellite cells. Cells from 7 participants were used in this study (3 men, 4 women, age 51.0 ± 2.4 years, BMI 29.9 ± 2.4 kg/m^2^).

### Cell culture

Human satellite cells were isolated from muscle biopsy samples as previously described [18]. Isolation of satellite cells was performed based on the method of Henry et al. [19] and Gaster et al. [20]. In brief, satellite cells were isolated from muscle biopsies, decontaminated of fibroblasts and grown to 3–6 passages. The isolated cells were cultured and proliferated in DMEM-GlutaMAX (5.5 mM glucose) supplemented with 10% FBS, HEPES (25 mM), gentamicin (50 ng/ml), penicillin (25 IU), streptomycin (25 μg/ml), amphotericin B (1.25 μg/ml), hEGF (10 ng/ml), dexamethasone (0.39 μg/ml) and 0.05% BSA (proliferation medium). The 2D muscle cells and myospheres were cultured as previously described [16]. In brief, for the myospheres formation (4–8 h), around 3 × 10^4^ myoblasts per well were seeded in proliferation medium as a suspension and aggregated by natural sedimentation in a Nunclon™ Sphera™ 96-well plate with ultra-low attachment treatment and a U-shaped well bottom. 24 h after, proliferation media was changed to DMEM-Glutamax™ (5.5 mmol/L glucose) containing 2% FBS, 25 p.m. insulin, HEPES (25 mmol/l), amphotericin B (1.25 μg/ml), gentamicin (50 ng/ml), penicillin (25 IU), and streptomycin (25 μg/ml) (differentiation medium) and the differentiation phase was carried out for 10 days. For 2D cultures from the same cells, see [8].

### Substrate oxidation assay

**Fig. 1:**
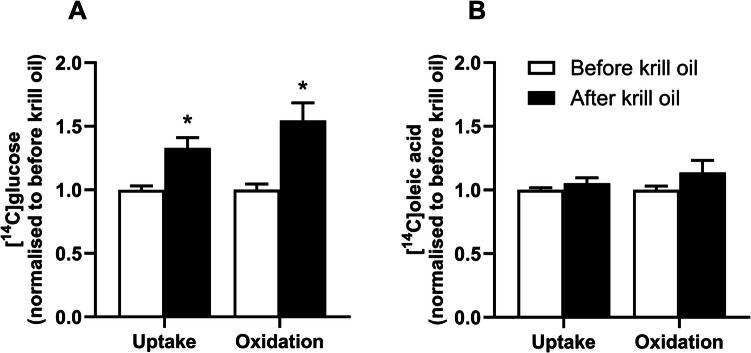
Effect of in vivo krill oil supplementation on glucose and oleic acid metabolism in myospheres. Myoblasts from 7 donors obtained before and after krill oil intervention were cultured as myospheres and differentiated for 10 days before the cells were incubated with [U-^14^C]glucose or [1-^14^C]oleic acid for 4 h. The figures show glucose uptake and oxidation related to before krill oil intervention **A** and oleic acid uptake and oxidation related to before krill oil intervention **B**, presented as mean ± SEM of 7 donors, each with 4–5 biological replicates. Absolute values before intervention: glucose uptake 12.1 ± 0.9 nmol/mg protein, glucose oxidation 4.0 ± 0.5 nmol/mg protein, oleic acid uptake 11.7 ± 0.4 nmol/mg protein, and oleic acid oxidation 1.4 ± 0.1 nmol/mg protein. *p < 0.05, unpaired t-test. *P*-values in A: uptake *p* = 0.0003, oxidation *p* = 0.0003, in B: uptake *p* = 0.26, oxidation *p* = 0.17

After 10 days of differentiation, the myospheres were incubated with D-[^14^C(U)]glucose or [1-^14^C]OA as described [21]. In short, differentiation media was changed for 50 µl of either glucose radioactive substrate (0.5 μCi/ml, 200 μM prepared in DPBS with BSA (10 μM) and HEPES (10 mM)) or OA (0.5 μCi/ml, 100 µM prepared in DPBS with HEPES (10 mmol/l), L-carnitine (1 mM) and BSA (40 μM). Subsequently, 96 well plates were mounted with a 96-well UniFilter® microplate, previously activated by adding 1 M NaOH in the trapping device. After 4 h incubation, cells and myospheres were washed with PBS and harvested in 0.1 M NaOH. ^14^CO_2_ trapped in the filter, produced from the metabolized substrate, and cell-associated (CA) radioactivity from glucose or OA, were measured by addition of scintillation fluid and counted on a 2450 MicroBeta^2^ scintillation counter. The obtained data were normalized by the protein content measured by Bio-Rad protein assay using a VICTOR™ X4 Multilabel Plate Reader (PerkinElmer). The total uptake of each substrate was calculated as the sum of both ^14^CO_2_ and CA radioactivity (CO_2_ + CA). Values (in Fig. 1) are presented as mean ± SEM, and the statistical analyses (unpaired t-test) were performed using GraphPad Prism 10.2.0. *P* < 0.05 was considered significant.

### RNA isolation and high throughput RNA sequencing

The myospheres were pooled, washed with PBS, and sunk by centrifugation in a 1.5 ml Eppendorf microcentrifuge tube. 1 ml of TRIzol was added and the sample was homogenized by IKA® Ultra-Turrax® tissue homogenizer (Sigma-Aldrich, St. Louis, MO, US) for the complete RNA isolation. According to the manufacturer´s protocol, 0.2 ml of chloroform added directly to 1 ml of TRIzol was used for lysis and separation of the aqueous phase containing RNA. 0.5 ml of isopropanol was mixed to precipitate RNA. The RNA pellet was washed with 75% ethanol and resuspended in 15 µl of RNase-free water before the quantity and quality of the RNA were assessed.

The RNA library preparation and transcriptome sequencing processes were outsourced to Novogene Co., LTD. (Milton, United Kingdom), utilizing their Illumina platform for paired-end sequencing. The library preparation and RNA-seq analysis were carried out at Novogene Co., LTD. (Milton, United Kingdom). To identify differentially expressed genes Novomagic (https://magic.novogene.com) online platform was used. Differential expression analysis was performed using the DESeq2 R package (version 1.20.0). DESeq2 utilizes a model based on the negative binomial distribution for statistical analysis of digital gene expression data. The resulting *p*-values were adjusted using the Benjamini and Hochberg's approach to control the false discovery rate. Significantly differential expression was determined using thresholds of padj < 0.05 and |log2(fold-change)|> 0. For pathway enrichment analysis of differential expressed genes, Database for Annotation, Visualization and Integrated Discovery (DAVID) (https://david.ncifcrf.gov) was used. The sequence data are submitted to the Gene Expression Omnibus and are accessible through the identifier GSE291925 at http://www.ncbi.nlm.nih.gov/geo. For the myophere- 2D comparison, sequence data from [8], identifier GSE278505 have also been used.

### Proteomic analysis

Myospheres were cultured and differentiated for 10 days, washed in DPBS and spun down at 1000 rpm at 4 °C for 5 min. The cells were then snap frozen in liquid nitrogen and stored at −80 °C. The proteomic analysis, using LC–MS/MS, have been described previously [8, 22]. Raw files from LC–MS/MS analyses were submitted to MaxQuant software (ver 2.0.3.0) for protein identification and label-free quantification. Parameters were set as follow: Carbamidomethyl (C) was set as a fixed modification and protein N-acetylation and methionine oxidation as variable modifications. First search error window was 20 ppm and main search error 6 ppm. Trypsin without proline restriction enzyme option was used, with two allowed miscleavages. Minimal unique peptides were set to one, and FDR allowed was 0.01 (1%) for peptide and protein identification. The Uniprot human database was used. Generation of reversed sequences was selected to assign FDR rate. Known contaminants and reversed entries as provided by MaxQuant and identified in samples were excluded from further analysis. MaxQuant proteinGroup-file was further analyzed in Perseus software (ver 1.6.15.0). Normalised intensities (LFQ) were log10 transformed, data was filtered to have at least 3/5 valid values in at least one group, missing values were imputed from normal distribution using default settings and statistical analysis was done using paired t-test with *P* < 0.05 as the criteria. KEGG pathway analysis of differential regulated proteins was made using STRING version 12.0 (https://string-db.org/). The mass spectrometry proteomics data have been deposited to the ProteomeXchange Consortium via the PRIDE [23] partner repository with the dataset identifier PXD055840. For the myosphere and 2D comparison, mass spectrometry proteomics data from [8], data set identifier PXD052661 have also been used. For this comparison, we focused on proteins with the largest differences (i.e. proteins where at least 4 of the 5 donors had a signal in one group and 4 of 5 donors had no signal in the opposite group).

## Results

### In vivo krill oil supplementation increased glucose uptake and oxidation but had no effect on oleic acid metabolism in cultured myospheres

In 2D cultures from the same cells, myotubes obtained from biopsies taken after 7 weeks of krill oil intervention showed increased oleic acid oxidation, and no effect on glucose metabolism compared to myotubes from biopsies taken before the intervention [8]. To study whether 3D myospheres cultured from the same donors showed the same metabolic pattern, myospheres differentiated for 10 days were incubated for 4 h with [U-^14^C]glucose or [1-^14^C]oleic acid before uptake and oxidation of both radiolabelled substrates were measured.

As shown in Fig. 1, both glucose uptake and oxidation were increased in myospheres from cells obtained after the intervention compared to before (Fig. 1A), however no effects of in vivo krill oil intervention were found on oleic acid metabolism in myospheres (Fig. 1B).

### In vivo krill oil intervention changed global gene and protein expression in myospheres

**Fig. 2:**
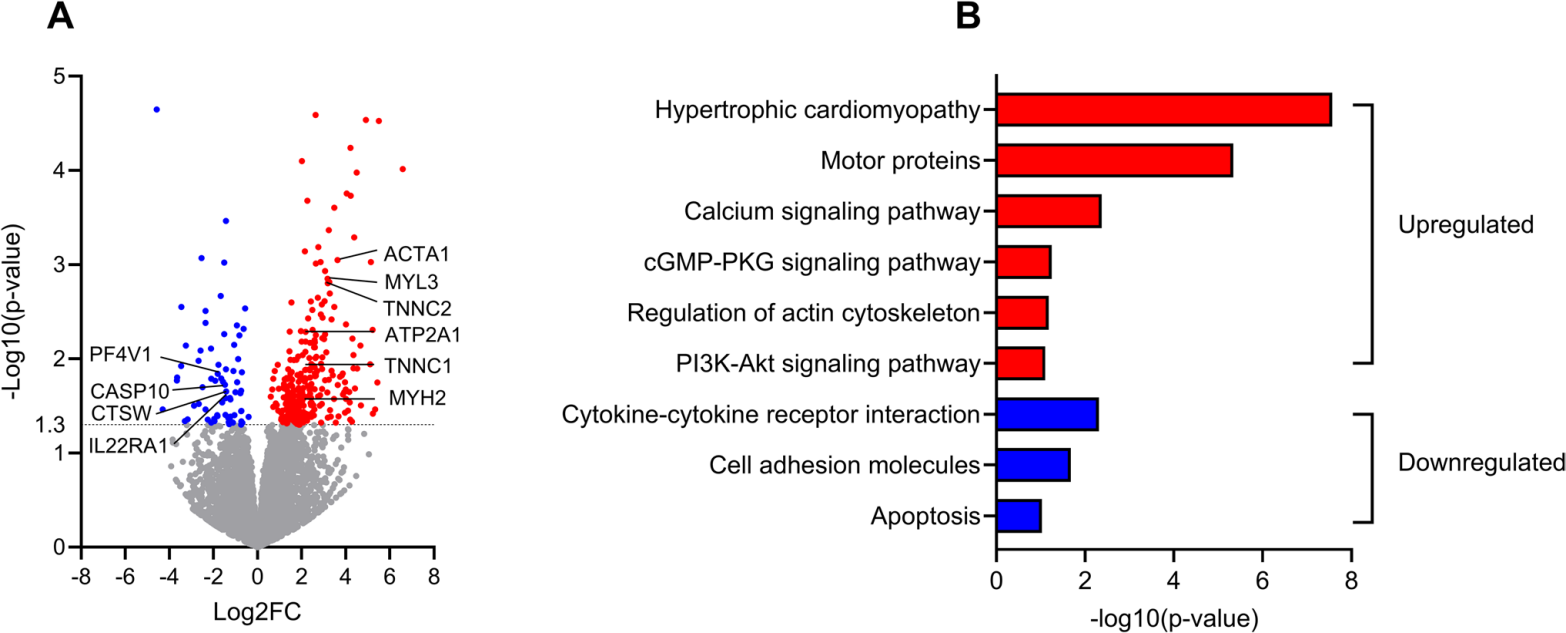
Effect of in vivo krill oil supplementation on gene expression in myospheres. Myospheres from myoblasts obtained before and after krill oil intervention, from 4 different donors, were analyzed. **A** Volcano plot displaying the differential gene expression between the control (before intervention) and krill oil-treated (after intervention) myospheres. Genes with significant changes in expression (*P* ≤ 0.05) are colored, downregulated: blue, upregulated: red. **B** KEGG pathway analysis of upregulated (red) and downregulated (blue) genes in myospheres after krill oil intervention made using Database for Annotation, Visualization and Integrated Discovery (DAVID) (https://david.ncifcrf.gov, accessed 03.06.24). The pathway enrichment analysis highlights the biological processes affected by the intervention. A selection of regulated pathways is shown. The *p*-values in B relate to the DAVID analyses. ACTA1, actin alpha skeletal muscle; ATP2A1, sarcoplasmic/endoplasmic reticulum calcium ATPase 1; CASP10, caspase-10; CTSW, cathepsin W; IL22RA1, interleukin-22 receptor subunit alpha-1; MYH2, myosin 2; MYL3, myosin light chain 3; PF4V1, platelet factor 4 variant; PI3K, phospatidylinosititol-3-kinase; PKG, cGMP-dependent protein kinase 1; TNNC1, troponin C, slow skeletal and cardiac muscles; TNNC2, troponin C, skeletal muscle

**Fig. 3:**
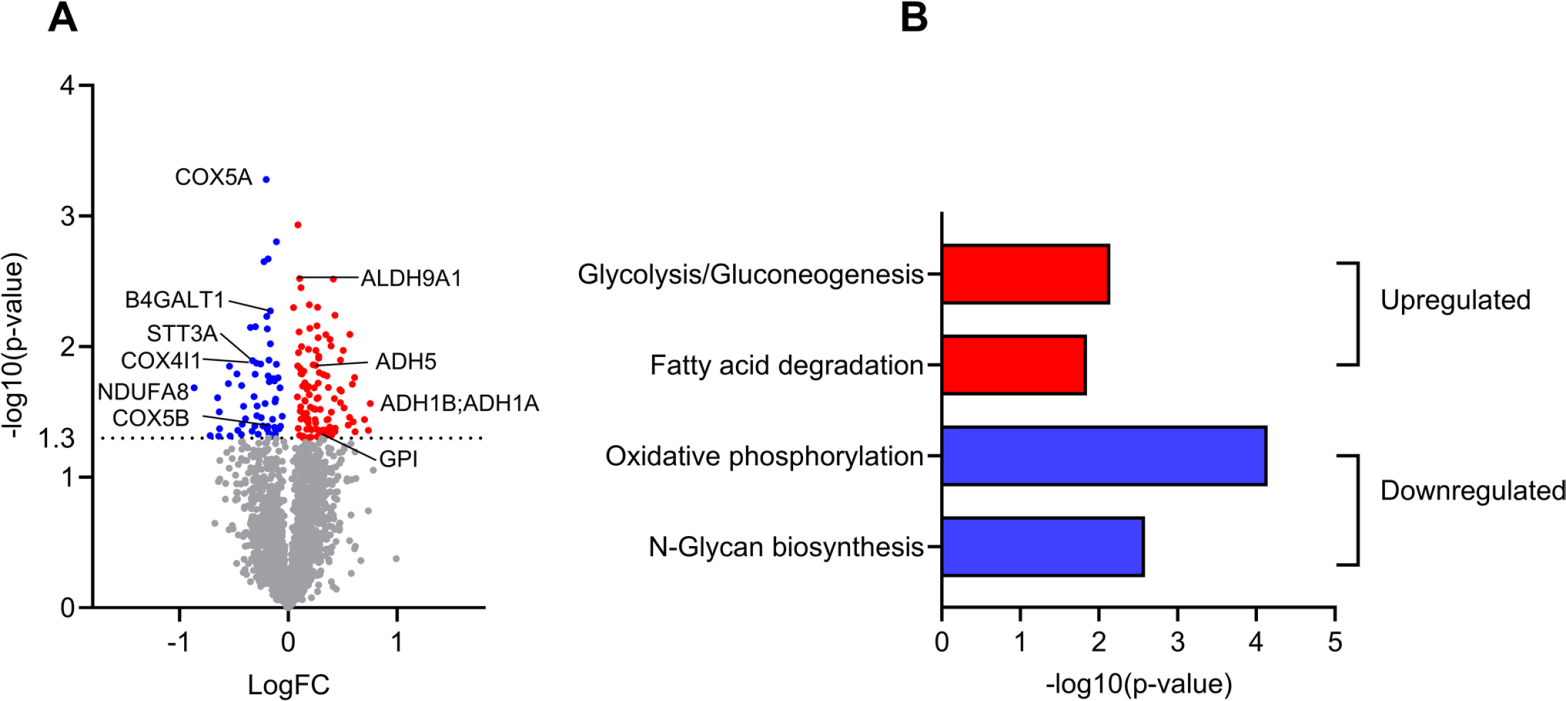
Effect of in vivo krill oil supplementation on protein expression in myospheres. Myospheres from myoblasts obtained before and after krill oil intervention, from 5 different donors, were analyzed. **A** Volcano plot displaying the differential protein expression between the control (before intervention) and krill oil-treated (after intervention) myospheres. Genes with significant changes in expression (*P* ≤ 0.05) are colored, downregulated: blue, upregulated: red. **B** KEGG pathway analysis of upregulated (red) and downregulated (blue) proteins in myospheres after krill oil intervention made using STRING version 12.0 (https://string-db.org/, accessed 04.09.24). The pathway enrichment analysis highlights the biological processes affected by the intervention. A selection of regulated pathways is shown. The *p*-values in B relate to the STRING analyses. ADH1A, alcohol dehydrogenase 1A; ADH1B, alcohol dehydrogenase 1B; ADH5, alcohol dehydrogenase class-3; ALDH9A1, 4-trimethylaminobutyraldehyde dehydrogenase; B4GALT1, beta-1,4-galactosyltransferase 1; COX4I1, cytochrome c oxidase subunit 4 isoform; COX5 A, cytochrome c oxidase subunit 5 A; COX5B, cytochrome c oxidase subunit 5B; GPI, Glucose-6-phosphate isomerase; NDUFA8, NADH dehydrogenase 1 alpha subcomplex subunit 8; STT3A, dolichyl-diphosphooligosaccharide-protein glycosyltransferase subunit STT3 A

To better understand the impact of krill oil intervention on myospheres, we conducted transcriptomic and proteomic analyses before and after the krill oil supplementation comparing gene (Fig. 2) and protein (Fig. 3) expression profiles. Due to technical reasons, only four and five of the donors were successfully analyzed for gene and protein expression, respectively.

The transcriptomic analysis identified 16,890 protein-coding gene transcripts within the myospheres. Among these, 267 gene transcripts were found to be significantly upregulated by krill oil supplementation, while 68 gene transcripts were significantly downregulated (Fig. 2A) (Supplementary Table 1). Pathway analysis using Database for Annotation, Visualization and Integrated Discovery (DAVID) (https://david.ncifcrf.gov, accessed 03.06.24) [24, 25] showed some interesting KEGG pathways where upregulated and downregulated genes were involved (Fig. 2B). The hypertrophic cardiomyopathy pathway contains upregulated genes like sarcoplasmic/endoplasmic reticulum calcium ATPase 1 (ATP2 A1), myosin light chain 3 (MYL3) and troponin C, skeletal muscle (TNNC2). Proteins involved in the motor proteins pathway like MYL3, TNNC2, actin alpha, skeletal muscle (ACTA1), myosin 2 (MYH2), myosin light chain 3 (MYL3) and troponin C, slow skeletal and cardiac muscles (TNNC1) were also upregulated (Supplementary Fig. 1, figure used with permission from the KEGG database project [26]). Other upregulated genes were part of pathways like calcium signaling, cGMP-PKG (cGMP-dependent protein kinase 1), actin cytoskeleton regulation, and PI3K-Akt (phospatidylinosititol-3-kinase-Akt) signaling pathways. In comparison, pathways like hypertrophic cardiomyopathy and motor proteins were not upregulated in the transcriptomic analysis of 2D cultures from the same clinical study [8].

Among the downregulated genes, pathways of cytokine-cytokine receptor interaction (interleukin-22 receptor subunit alpha-1 (IL22RA1)), platelet factor 4 variant (PF4V1), cell adhesion molecules, and apoptosis (e.g. caspase- 10 (CASP10) and cathepsin W (CTSW)) were identified.

The proteomic analysis identified 3381 proteins, of which 104 were upregulated and 61 were downregulated (Fig. 3A and Supplementary Table 2). Pathway analysis using STRING (https://string-db.org/, accessed 04.09.24) showed some KEGG pathways of interest for upregulated and downregulated proteins (Fig. 3B). Upregulated proteins including e.g. alcohol dehydrogenase 1A (ADH1A) and 1B (ADH1B), alcohol dehydrogenase class-3 (ADH5), and 4-trimethylaminobutyraldehyde dehydrogenase (ALDH9A1) were both in the glycolysis/gluconeogenesis and fatty acid degradation pathways. Glucose- 6-phosphate isomerase (GPI) was also in the pathway glycolysis/gluconeogenesis. In contrast, downregulated proteins were e.g. part of oxidative phosphorylation pathway (cytochrome c oxidase subunit 4 isoform (COX4I1), subunit 5 A (COX4A), subunit 5B (COX5B), and NADH dehydrogenase 1 alpha subcomplex subunit 8 (NDUFA8) and the N-glycan biosynthesis pathway (beta-1,4-galactosyltransferase 1 (B4GALT1) and dolichyl-diphosphooligosaccharide-protein glycosyltransferase subunit STT3A).

Comparing the effect of krill oil intervention in myospheres and 2D cultures [8], 20 protein-coding genes in the transcriptomic data were similarly regulated, 19 upregulated and 1 downregulated (Supplementary Table 3), however, none of the proteins in the proteomic data were similarly regulated.

### Comparison of myospheres and 2D cultures at basal level

**Fig. 4:**
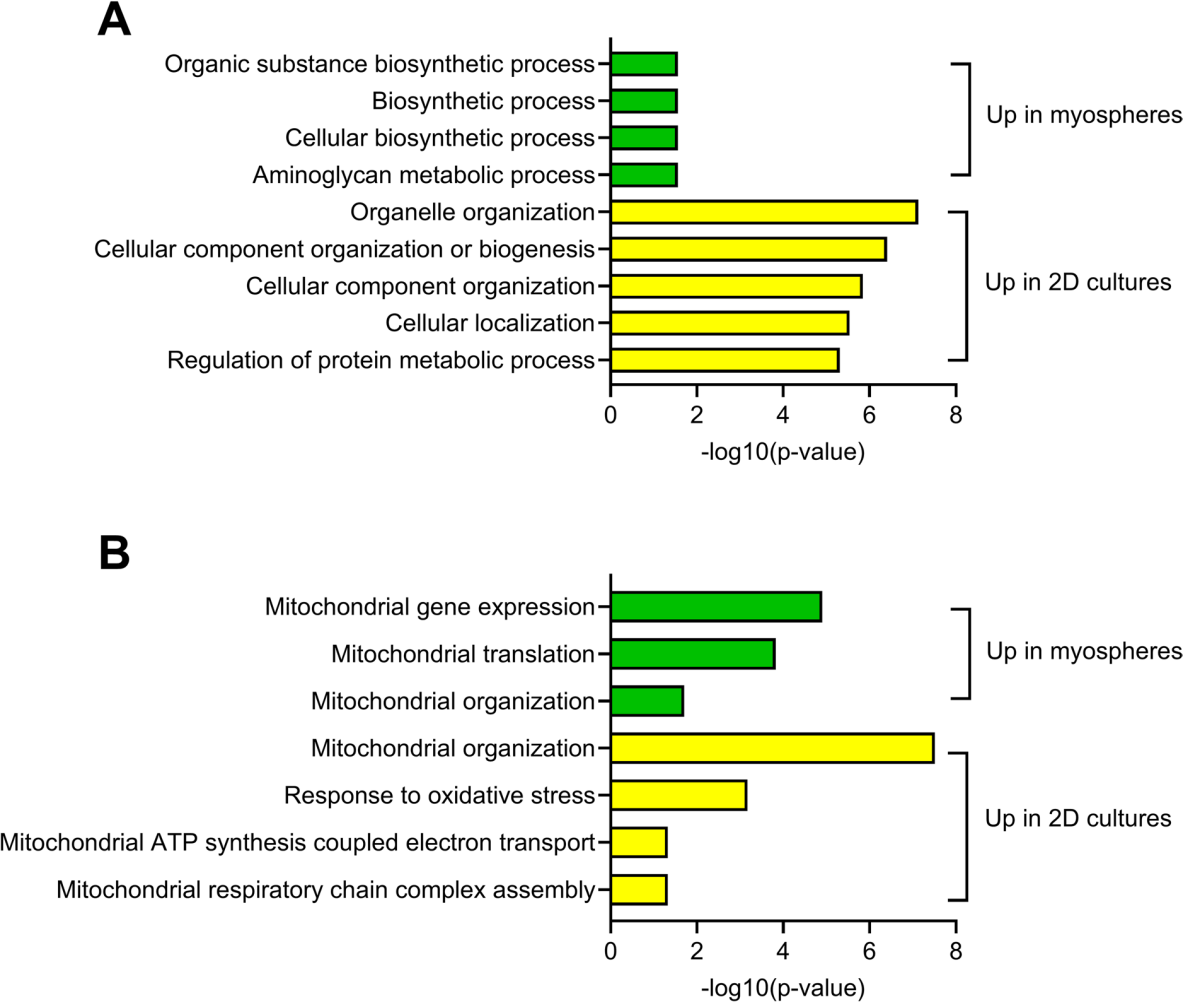
Comparison of proteins in myospheres and 2D myotube cultures at baseline. Myoblasts from five donors obtained before the krill oil intervention cultured as myospheres and differentiated for 10 days, or cultured as 2D cultures differentiated for 7 days, were analyzed. The figure shows selected biological processes (Gene Ontology) of all **A** or mitochondria-related **B** proteins (selected using human MitoCarta 3.0, https://www.broadinstitute.org/mitocarta, accessed 18.10.24) that were expressed in at least 4 of the 5 donors in one group and 4 of 5 donors had no signal in the opposite group, analyzed by STRING version 12.0 (https://string-db.org/, accessed 05.11.25 (**A**) or 18.10.24 (**B**)). The *p*-values relate to the STRING analyses

To further compare the myospheres and 2D cultures, the proteomic data from muscle cells at the basal level (before krill oil intervention) from five donors were compared. A new in silico analysis was performed, using data from two different proteomic analyses (3D and 2D cultures from the same donors) where it was focused on proteins with the largest differences (on–off differences, i.e. proteins where at least 4 of the 5 donors had a signal in one group and 4 of 5 donors had no signal in the opposite group). In this analysis, 96 proteins were found in the myospheres and 533 proteins were found in the 2D cultures (Supplementary Table 4). These proteins were analyzed by STRING version 12.0 (https://string-db.org/, accessed 05.11.24). Four biological processes (Gene Ontology) were found for the myospheres (Fig. 4A), while for the 2D cultures, several biological processes were found, as expected from the large number of proteins exclusively detected in these cultures. The five biological processes with the lowest *p*-value are shown in Fig. 4A. Three of four of fhe biological processes found for the 3D cultures were biosynthetic processes (cellular biosynthetic process, biosynthetic process, organic substance biosynthetic process) sharing several proteins, e.g. nicotinamide/nicotinic acid mononucleotide adenylyltransferase 1 (NMNAT1), prostaglandin G/H synthase 1 (PTGS1) and soluble calcium-activated nucleotidase 1 (CANT1). The biological process with the lowest *p*-value for 2D cultures was organelle organisation, covering e.g. proteins like NAD-dependent protein deacetylase sirtuin-2 (SIRT2), cohesin subunit SA-1 (STAG1) and nuclear pore complex protein Nup153 (NUP153).

Compared with human MitoCarta 3.0 (https://www.broadinstitute.org/mitocarta, accessed 18.10.24 [27]), 15 of the proteins in the myosphere group and 31 of the proteins in the 2D myotube group were related to the mitochondria. The mitochondria-related proteins were analyzed by STRING version 12.0 (https://string-db.org/, accessed 18.10.24). Biological processes (Gene Ontology) are shown in Fig. 4B, showing that in myospheres, mitochondrial gene expression and translation dominated (proteins involved are e.g. mitochondrial ribosomal proteins L13 (MRPL30), mitochondrial ribosomal proteins L30 (MRPL30), S24 (MRPS43), S23 (MRPS23), and S33 (MRPS33)), while in 2D cultures, mitochondrial organization (e.g. NADH dehydrogenase [ubiquinone] 1 alpha subcomplex assembly factors 4 (NDUFAF4), 2 (NDUFAF2), NADH dehydrogenase [ubiquinone] iron-sulfur protein 4 (NDUFS4), and cytochrome c oxidase subunit 7 A1, mitochondrial (COX7A1)) and response to oxidative stress (e.g. NADH-ubiquinone oxidoreductase chain 3 (MT-ND3)) were more important. Interestingly, pyruvate dehydrogenase kinase isozyme 1, mitochondrial (PDK1) was found in myospheres and not in the 2D cultures (Supplementary Table 4).

## Discussion

In this study, we have examined the effects of 7 weeks of in vivo krill oil supplementation in a myosphere model in vitro, using myoblasts isolated from biopsies obtained before and after the intervention. In contrast to previous data found in 2D myotube cultures, in vivo intervention with krill oil increased glucose metabolism in 3D myospheres, while no effect was observed on fatty acid metabolism. Transcriptomic analysis comparing myospheres produced from myoblasts obtained before and after krill oil supplementation identified differentially expressed genes associated with e.g. motor proteins and hypertrophy pathways. Interestingly, those pathways were not regulated in 2D cultures using cells from the same donors [8]. Proteomic analysis demonstrated upregulation of proteins involved in glycolysis/gluconeogenesis and fatty acid degradation in myospheres. In addition, comparison of proteins expressed exclusively either in myosphere or 2D cultures in the basal state (before the intervention) showed that the myospheres expressed proteins related to biosynthetic processes and mitochondrial protein expression and translation, while the 2D cultures expressed proteins related to organization of organelles, cellular components and mitochondria, as well as response to oxidative stress.

Krill oil is derived from Antarctic krill, and as a dietary supplement, krill oil may have beneficial effects on several aspects of human health (reviewed in [1, 2]). In myospheres, our 3D cell model, in vivo krill oil supplement caused upregulation of genes involved in several pathways like muscle hypertrophy and motor proteins. This was not reflected in the proteomic data, possible due to both the fact that the untargeted proteomic analysis used in this study do not detect all proteins. However, the upregulation of pathways of hypertropy and motor proteins is in accordance with the increased skeletal muscle strength, power and recovery of injury found after in vivo krill oil supplementation in some human studies [6, 7]. In addition, krill ethanol extracts have been shown to increase muscle regeneration and improve muscle function in a mouse model [28].

Krill oil contains omega-3 polyunsaturated fatty acids (PUFA), palmitoleic acid and the antioxidant astaxanthin, each of which could potentially be responsible or somehow contribute to the effects found in skeletal muscle. Several studies have explored the effects of these components on skeletal muscle mass and function, partly with varying results. Supplementation of omega-3 PUFA (4 g/day) for 8 weeks stimulated muscle protein synthesis in older (≥ 65 years) men and women [29], and the same dose for 6 months increased muscle mass and muscle function, compared to corn oil, in men and women 60–85 years [30]. In line with this, omega-3 PUFA (3.9 g/day) supplementation for 16 weeks increased skeletal muscle mitochondrial and myofibrillar protein synthesis in older (65–85 years) human subjects [31]. A meta-analysis of 10 randomized clinical studies [32] evaluated the potential effect of omega-3 PUFA, either in diet or as a supplement, in sarcopenia-related performances in elderly (≥ 60 years) subjects. Muscle mass was significantly increased, however, muscle strength seemed not to be increased by omega-3 PUFA [32]. Another meta-analysis of five studies including 488 participants ≥ 65 years also concluded that omega-3 PUFA supplementation might increase muscle mass but had no effect on muscle strength or functional abilities [33]. In contrast, a third meta-analysis of 14 clinical studies, including 1433 men and women, adults and old adults, found no significant effect of omega-3 PUFA compared to placebo on muscle mass, however, a small but significant increase in muscle strength was found [34]. Palmitoleic acid is an unusual omega-7 monounsaturated fatty acid known to have several biological functions (reviewed in [35]), however, little is known regarding its effects on skeletal muscle. Astaxanthin was shown to protect against both cancer cachexia-induced atrophy [36] and sarcopenia associated with chronic obstructive pulmonary disease [37] in mice skeletal muscle. In clinical studies, astaxanthin has been shown to reduce muscle and exercise-induced damage (reviewed in [38]). In healthy older adults (65–85 years), astaxanthin supplementation increased fat oxidation and muscle endurance after 3 months of endurance training compared to training with placebo [39]. However, in resistant-trained males on an exercise-induced muscle damage protocol, astaxanthin supplement for four weeks did not affect markers of muscle damage or inflammation [40].

Previously, we showed that glucose metabolism (uptake and oxidation) was similar in myospheres and 2D myotube cultures [16]. In the current study, in vivo krill oil intervention induced an increase in these parameters in the myosphere model, in contrast to previously obtained data in a 2D myotube model [8]. No effects of krill oil supplementation on glucose homeostasis were reported in clinical studies either [1, 2], however, it has been demonstrated that astaxanthin reduced glucose concentration in subjects with diabetes [41]. Also, krill oil consumption improved glucose metabolism in mice [3–5]. The upregulated pathway of glycolysis/gluconeogenesis from the proteomic analysis may confirm the increased utilization of carbohydrates found in the myospheres.

Regarding lipid metabolism, our previous data showed that in vivo krill oil intervention induced an increase of oleic acid oxidation in the 2D myotube model [8], while no effect on oleic acid metabolism was observed in the myospheres. However, this effect might have been masked by the fact that myospheres already have higher lipid oxidative metabolism compared to the 2D myotube model under basal conditions [16].

Comparing the results from myospheres in this study versus 2D myotube cultures from the same donors [8], this study emphasizes the contrast between the utility of 2D and 3D myotube models when analyzing the effect of various interventions in cell metabolism. Thus, the effects of in vivo krill oil intervention on myotubes in vitro analyzed by both measurements of fuel metabolism and gene/protein expression differed between the two cell models. As 2D is the most used model, it is well known that these myotube cultures have several limitations compared to the in vivo situation, e.g. many genes are expressed to a lower extent in 2D cultures compared to biopsies [9]. 3D muscle cell models may be a more mature and physiological relevant model for skeletal muscle in vivo [10–13], e.g. the mRNA expression of the fiber type markers myosin heavy chains 2 (*MYH2*) and 7 (*MYH7*) was higher in 3D compared to 2D cell cultures [16], although many in vivo factors such as direct and indirect contacts with other cell types and muscle contraction are also missing in this cell model. Thus, the 2D and 3D cell models may be complementary and both cell models might possibly provide valuable insights depending on the specific biological question being addressed.

Moreover, at the basal level before krill oil intervention, the two cell models showed, to some degree, different patterns of protein expression. The number of proteins solely expressed in myospheres (96 identities) was much lower than the number of proteins solely expressed in the 2D cultures (533 identities). In addition, the myospheres expressed, amongst others, proteins related to biosynthetic processes, while the 2D cultures e.g. expressed some proteins related to organelle and cellular component organization. The observed discrepancies between the models suggest distinct regulatory mechanisms, likely influenced by differences in cellular organization, extracellular matrix interactions, and metabolic adaptations.

When the differentially expressed proteins were limited to mitochondria-related proteins, mitochondrial protein expression and translation dominated in myospheres, while in 2D cultures, proteins involved in mitochondrial organization and response to oxidative stress were more important. Interestingly, pyruvate dehydrogenase kinase isozyme 1, mitochondrial (PDK1), which plays an important role in protein metabolism and cell growth [42] was found in the myospheres and not in the 2D cultures.

The findings of this study indicate that different models may yield varying outcomes, which may or may not be translated in clinical settings depending on the specific study goals. More research comparing these two skeletal muscle cell models is needed to conclude the significance of the observed differences in the present study, to demonstrate whether the myosphere model offers advantages over the 2D cell model and how these models might be translated into potential therapeutic strategies and clinical practices.

## Conclusion

This study suggests that the metabolic effects of in vivo krill oil intervention in skeletal muscle were differently expressed in 3D and 2D cultures, and that the two cell models can provide complimentary information. While the 2D model might be a better cell model to study metabolic effects of e.g. in vivo krill oil interventions, the 3D model may possibly better reflect in vivo krill oil-induced changes in skeletal muscle performance. In addition, comparison of untreated myospheres and 2D myotube cultures showed different patterns of protein expression.

## Supplementary Information

Below is the link to the electronic supplementary material.

Supplementary file1 (TIF 1198 KB)

Supplementary file2 (DOCX 61 KB)

Supplementary file3 (XLSX 28 KB)

Supplementary file4 (DOCX 16 KB)

Supplementary file5 (XLSX 38 KB)

## Data Availability

The data presented in the study are deposited in the Gene Expression Omnibus repository, accession number GSE291925 and GSE278505 and in the ProteomeXchange Consortium via the PRIDE, accession number PXD055840.

## References

[1] Ulven SM. Chapter 30 - Metabolic effects of krill oil. In: Raatz S, Bibus D, editors. Fish and Fish Oil in Health and Disease Prevention, Elsevier Inc.; 2016; 333–339

[2] Huang H, Liao D, He B, Zhou G, Cui Y. Clinical effectiveness of krill oil supplementation on cardiovascular health in humans: An updated systematic review and meta-analysis of randomized controlled trials. Diabetes Metab Syndr. 2023. 10.1016/j.dsx.2023.102909. 38039646 10.1016/j.dsx.2023.102909

[3] Sun D, Zhang L, Chen H, Feng R, Cao P, Liu Y. Effects of Antarctic krill oil on lipid and glucose metabolism in C57BL/6J mice fed with high fat diet. Lipids Health Dis. 2017. 10.1186/s12944-017-0601-8. 29157255 10.1186/s12944-017-0601-8 PMC5697064

[4] Rossmeisl M, Pavlisova J, Bardova K, Kalendova V, Buresova J, Kuda O, Kroupova P, Stankova B, Tvrzicka E, Fiserova E, Horakova O, Kopecky J. Increased plasma levels of palmitoleic acid may contribute to beneficial effects of Krill oil on glucose homeostasis in dietary obese mice. Biochim. Biophys. Acta Mol. Cell Biol Lipids. 2020; 10.1016/j.bbalip.2020.158732 10.1016/j.bbalip.2020.158732 32371092

[5] Hwang SM, Kim YU, Kim JK, Chun YS, Kwon YS, Ku SK, Song CH. Preventive and Therapeutic Effects of Krill Oil on Obesity and Obesity-Induced Metabolic Syndromes in High-Fat Diet-Fed Mice. Mar Drugs. 2022. 10.3390/md20080483. 36005486 10.3390/md20080483 PMC9410137

[6] Alkhedhairi SA, Aba Alkhayl FF, Ismail AD, Rozendaal A, German M, MacLean B, Johnston L, Miller A, Hunter AM, Macgregor LJ, Combet E, Quinn TJ, Gray SR. The effect of krill oil supplementation on skeletal muscle function and size in older adults: A randomised controlled trial. Clin Nutr. 2022. 10.1016/j.clnu.2022.04.007. 35504165 10.1016/j.clnu.2022.04.007

[7] Yang S, He Q, Shi L, Wu Y. Impact of Antarctic krill oil supplementation on skeletal muscle injury recovery after resistance exercise. Eur J Nutr. 2023. 10.1007/s00394-022-03077-6. 36566465 10.1007/s00394-022-03077-6

[8] Katare PB, Dalmao-Fernandez A, Mengeste AM, Navabakbar F, Hamarsland H, Ellefsen S, Berge RK, Bakke HG, Nyman TA, Kase ET, Rustan AC, Thoresen GH. Krill oil supplementation *in vivo* promotes increased fuel metabolism and protein synthesis in cultured human skeletal muscle cells. Front Nutr. 2024. 10.3389/fnut.2024.1452768. 39555189 10.3389/fnut.2024.1452768 PMC11565515

[9] Aas V, Bakke SS, Feng YZ, Kase ET, Jensen J, Bajpeyi S, Thoresen GH, Rustan AC. Are cultured human myotubes far from home? Cell Tissue Res. 2013. 10.1007/s00441-013-1655-1. 23749200 10.1007/s00441-013-1655-1

[10] Dessauge F, Schleder C, Perruchot MH, Rouger K. 3D in vitro models of skeletal muscle: myopshere, myobundle and bioprinted muscle construct. Vet Res. 2021. 10.1186/s13567-021-00942-w. 34011392 10.1186/s13567-021-00942-w PMC8136231

[11] Carraro E, Rossi L, Maghin E, Canton M, Piccoli M. 3D in vitro Models of Pathological Skeletal Muscle: Which Cells and Scaffolds to Elect? Front Bioeng Biotechnol. 2022. 10.3389/fbioe.2022.941623. 35898644 10.3389/fbioe.2022.941623 PMC9313593

[12] Wang K, Smith SH, Iijima H, Hettinger ZR, Mallepally A, Shroff SG, Ambrosio F. Bioengineered 3D Skeletal Muscle Model Reveals Complement 4b as a Cell-Autonomous Mechanism of Impaired Regeneration with Aging. Adv Mater. 2023. 10.1002/adma.202207443. 36650030 10.1002/adma.202207443 PMC13175247

[13] Kim H, Kim GS, Hyun SH, Kim E. Advancements in 2D and 3D In Vitro Models for Studying Neuromuscular Diseases. Int J Mol Sci. 2023. 10.3390/ijms242317006. 38069329 10.3390/ijms242317006 PMC10707046

[14] van der Wal E, Iuliano A, In’t Groen SLM, Bholasing AP, Priesmann D, Sharma P, den Hamer B, Saggiomo V, Krüger M, Pijnappel WWMP, de Greef JC. Highly contractile 3D tissue engineered skeletal muscles from human iPSCs reveal similarities with primary myoblast-derived tissues. Stem Cell Reports. 2023;18(10):1954–71. 37774701 10.1016/j.stemcr.2023.08.014 PMC10656354

[15] Pinton L, Khedr M, Lionello VM, Sarcar S, Maffioletti SM, Dastidar S, Negroni E, Choi S, Khokhar N, Bigot A, Counsell JR, Bernardo AS, Zammit PS, Tedesco FS. 3D human induced pluripotent stem cell-derived bioengineered skeletal muscles for tissue, disease and therapy modeling. Nat Protoc. 2023. 10.1038/s41596-022-00790-8. 36792780 10.1038/s41596-022-00790-8

[16] Dalmao-Fernandez A, Aizenshtadt A, Bakke HG, Krauss S, Rustan AC, Thoresen GH, Kase ET. Development of three-dimensional primary human myospheres as culture model of skeletal muscle cells for metabolic studies. Front Bioeng Biotechnol. 2023. 10.3389/fbioe.2023.1130693. 37034250 10.3389/fbioe.2023.1130693 PMC10076718

[17] Ottestad I, Vogt G, Retterstøl K, Myhrstad MC, Haugen JE, Nilsson A, Ravn-Haren G, Nordvi B, Brønner K, Andersen LF, Holven KB, Ulven SM. Oxidised fish oil does not influence established markers of oxidative stress in healthy human subjects: A randomised controlled trial. Br J Nutr. 2012. 10.1017/S0007114511005484. 22136711 10.1017/S0007114511005484

[18] Lund J, Helle SA, Li Y, Løvsletten NG, Stadheim HK, Jensen J, Kase ET, Thoresen GH, Rustan AC. Higher lipid turnover and oxidation in cultured human myotubes from athletic versus sedentary young male subjects. Sci Rep. 2018. 10.1038/s41598-018-35715-7. 30510272 10.1038/s41598-018-35715-7 PMC6277406

[19] Henry RR, Abrams L, Nikoulina S, Ciaraldi TP. Insulin action and glucose metabolism in nondiabetic control and NIDDM subjects. Comparison using human skeletal muscle cell cultures. Diabetes. 1995;44(8):936–46. 10.2337/diab.44.8.936. 7622000 10.2337/diab.44.8.936

[20] Gaster M, Kristensen SR, Beck-Nielsen H, Schrøder HD. A cellular model system of differentiated human myotubes. APMIS. 2001. 10.1034/j.1600-0463.2001.d01-140.x. 11900052 10.1034/j.1600-0463.2001.d01-140.x

[21] Wensaas AJ, Rustan AC, Lövstedt K, Kull B, Wikström S, Drevon CA, Hallén S. Cell-based multiwell assays for the detection of substrate accumulation and oxidation. J Lipid Res. 2007. 10.1194/jlr.D600047-JLR200. 17213484 10.1194/jlr.D600047-JLR200

[22] Stevanovic S, Dalmao-Fernandez A, Mohamed D, Nyman TA, Kostovski E, Iversen PO, Savikj M, Nikolic N, Rustan AC, Thoresen GH, Kase ET. Time-dependent reduction in oxidative capacity among cultured myotubes from spinal cord injured individuals. Acta Physiol. (Oxf). 2024; 10.1111/apha.14156 10.1111/apha.14156 38711362

[23] Perez-Riverol Y, Csordas A, Bai J, Bernal-Llinares M, Hewapathirana S, Kundu DJ, Inuganti A, Griss J, Mayer G, Eisenacher M, Pérez E, Uszkoreit J, Pfeuffer J, Sachsenberg T, Yilmaz S, Tiwary S, Cox J, Audain E, Walzer M, Jarnuczak AF, Ternent T, Brazma A, Vizcaíno JA. The PRIDE database and related tools and resources in 2019: improving support for quantification data. Nucleic Acids Res. 2019; 10.1093/nar/gky1106 10.1093/nar/gky1106 PMC6323896 30395289

[24] Huang DW, Sherman BT, Lempicki RA. Systematic and integrative analysis of large gene lists using DAVID Bioinformatics Resources. Nature Protoc. 2009. 10.1038/nprot.2008.211. 10.1038/nprot.2008.211 19131956

[25] Sherman BT, Hao M, Qiu J, Jiao X, Baseler MW, Lane HC, Imamichi T, Chang W. DAVID: a web server for functional enrichment analysis and functional annotation of gene lists (2021 update). Nucleic Acids Res. 2022; 10.1093/nar/gkac194 10.1093/nar/gkac194 PMC9252805 35325185

[26] Kanehisa M, Furumichi M, Sato Y, Kawashima M, Ishiguro-Watanabe M. KEGG for taxonomy-based analysis of pathways and genomes. Nucleic Acids Res. 2023. 10.1093/nar/gkac963. 36300620 10.1093/nar/gkac963 PMC9825424

[27] Rath S, Sharma R, Gupta R, Ast T, Chan C, Durham TJ, Goodman RP, Grabarek Z, Haas ME, Hung WHW, Joshi PR, Jourdain AA, Kim SH, Kotrys AV, Lam SS, McCoy JG, Meisel JD, Miranda M, Panda A, Patgiri A, Rogers R, Sadre S, Shah H, Skinner OS, To TL, Walker MA, Wang H, Ward PS, Wengrod J, Yuan CC, Calvo SE, Mootha VK. MitoCarta3.0: an updated mitochondrial proteome now with sub-organelle localization and pathway annotations. Nucleic Acids Res. 2021; 10.1093/nar/gkaa1011 10.1093/nar/gkaa1011 PMC7778944 33174596

[28] Lee S, Baek M-O, Khaliq SA, Parveen A, Kim SY, Kim J-H, Kim I-C, Yoon M-S. Antarctic krill extracts enhance muscle regeneration and muscle function via mammalian target of rapamycin regulation. J Funct Foods. 2023. 10.1016/j.jff.2023.105483.

[29] Smith GI, Atherton P, Reeds DN, Mohammed BS, Rankin D, Rennie MJ, Mittendorfer B. Dietary omega-3 fatty acid supplementation increases the rate of muscle protein synthesis in older adults: a randomized controlled trial. Am J Clin Nutr. 2011. 10.3945/ajcn.110.005611. 21159787 10.3945/ajcn.110.005611 PMC3021432

[30] Smith GI, Julliand S, Reeds DN, Sinacore DR, Klein S, Mittendorfer B. Fish oil-derived n-3 PUFA therapy increases muscle mass and function in healthy older adults. Am J Clin Nutr. 2015. 10.3945/ajcn.114.105833. 25994567 10.3945/ajcn.114.105833 PMC4480667

[31] Lalia AZ, Dasari S, Robinson MM, Abid H, Morse DM, Klaus KA, Lanza IR. Influence of omega-3 fatty acids on skeletal muscle protein metabolism and mitochondrial bioenergetics in older adults. Aging (Albany NY) 2017; 10.18632/aging.101210. 10.18632/aging.101210 PMC5425117 28379838

[32] Huang YH, Chiu WC, Hsu YP, Lo YL, Wang YH. Effects of Omega-3 Fatty Acids on Muscle Mass, Muscle Strength and Muscle Performance among the Elderly: A Meta-Analysis. Nutrients. 2020. 10.3390/nu12123739. 33291698 10.3390/nu12123739 PMC7761957

[33] Timraz M, Binmahfoz A, Quinn TJ, Combet E, Gray SR. The Effect of Long Chain n-3 Fatty Acid Supplementation on Muscle Strength in Older Adults: A Systematic Review and Meta-Analysis. Nutrients. 2023. 10.3390/nu15163579. 37630768 10.3390/nu15163579 PMC10458650

[34] Santo André HC, Esteves GP, Barreto GHC, Longhini F, Dolan E, Benatti FB. The Influence of n-3PUFA Supplementation on Muscle Strength, Mass, and Function: A Systematic Review and Meta-Analysis. Adv Nutr. 2023. 10.1016/j.advnut.2022.11.005. 36811583 10.1016/j.advnut.2022.11.005 PMC10103001

[35] Hu W, Fitzgerald M, Topp B, Alam M, O’Hare TJ. A review of biological functions, health benefits, and possible de novo biosynthetic pathway of palmitoleic acid in macadamia nuts. J Funct Foods. 2019. 10.1016/j.jff.2019.103520.

[36] Yu X, Ren P, Yang R, Yue H, Tang Q, Xue C. Astaxanthin Ameliorates Skeletal Muscle Atrophy in Mice With Cancer Cachexia. Nutr Cancer. 2024. 10.1080/01635581.2024.2335584. 38567899 10.1080/01635581.2024.2335584

[37] Mano Y, Tsukamoto M, Wang KY, Nabeshima T, Kosugi K, Tajima T, Yamanaka Y, Suzuki H, Kawasaki M, Nakamura E, Zhou Q, Azuma K, Nakashima T, Tamura Y, Kozaki K, Nakazato K, Li YS, Kawai K, Yatera K, Sakai A. Oxidative stress causes muscle structural alterations via p38 MAPK signaling in COPD mouse model. J Bone Miner Metab. 2022. 10.1007/s00774-022-01371-1. 36163519 10.1007/s00774-022-01371-1

[38] Hecht KA, Schnackenberg J, Nair A, Lignell Å. Chapter 22 - Astaxanthin for improved muscle function and enhanced physical performance. In: Ravishankar GA, Rao AR, editors. Global Perspectives on Astaxanthin, Elsevier Inc 2021; 10.1016/B978-0-12-823304-7.00033-7

[39] Liu SZ, Valencia AP, VanDoren MP, Shankland EG, Roshanravan B, Conley KE, Marcinek DJ. Astaxanthin supplementation enhances metabolic adaptation with aerobic training in the elderly. Physiol Rep 2021; 10.14814/phy2.14887 10.14814/phy2.14887 PMC8191397 34110707

[40] Waldman HS, Bryant AR, Parten AL, Grozier CD, McAllister MJ. Astaxanthin Supplementation Does Not Affect Markers of Muscle Damage or Inflammation After an Exercise-Induced Muscle Damage Protocol in Resistance-Trained Males. J Strength Cond Res. 2023. 10.1519/JSC.0000000000004408. 36727984 10.1519/JSC.0000000000004408

[41] Medoro A, Intrieri M, Passarella D, Willcox DC, Davinelli S, Scapagnini G. Astaxanthin as a metabolic regulator of glucose and lipid homeostasis. J Funct Foods. 2024. 10.1016/j.jff.2023.105937.

[42] Kuramoto N, Nomura K, Kohno D, Kitamura T, Karsenty G, Hosooka T, Ogawa W. Role of PDK1 in skeletal muscle hypertrophy induced by mechanical load. Sci Rep. 2021. 10.1038/s41598-021-83098-z. 33568757 10.1038/s41598-021-83098-z PMC7876046

