# Supplementary figures and images for: The use of three-dimensional primary human myospheres to explore skeletal muscle effects of in vivo krill oil supplementation
Source: In Vitro Model. 2025 Apr 30;4(2):145–55. doi: 10.1007/s44164-025-00087-6 (PMC12283505; doi:10.1007/s44164-025-00087-6)

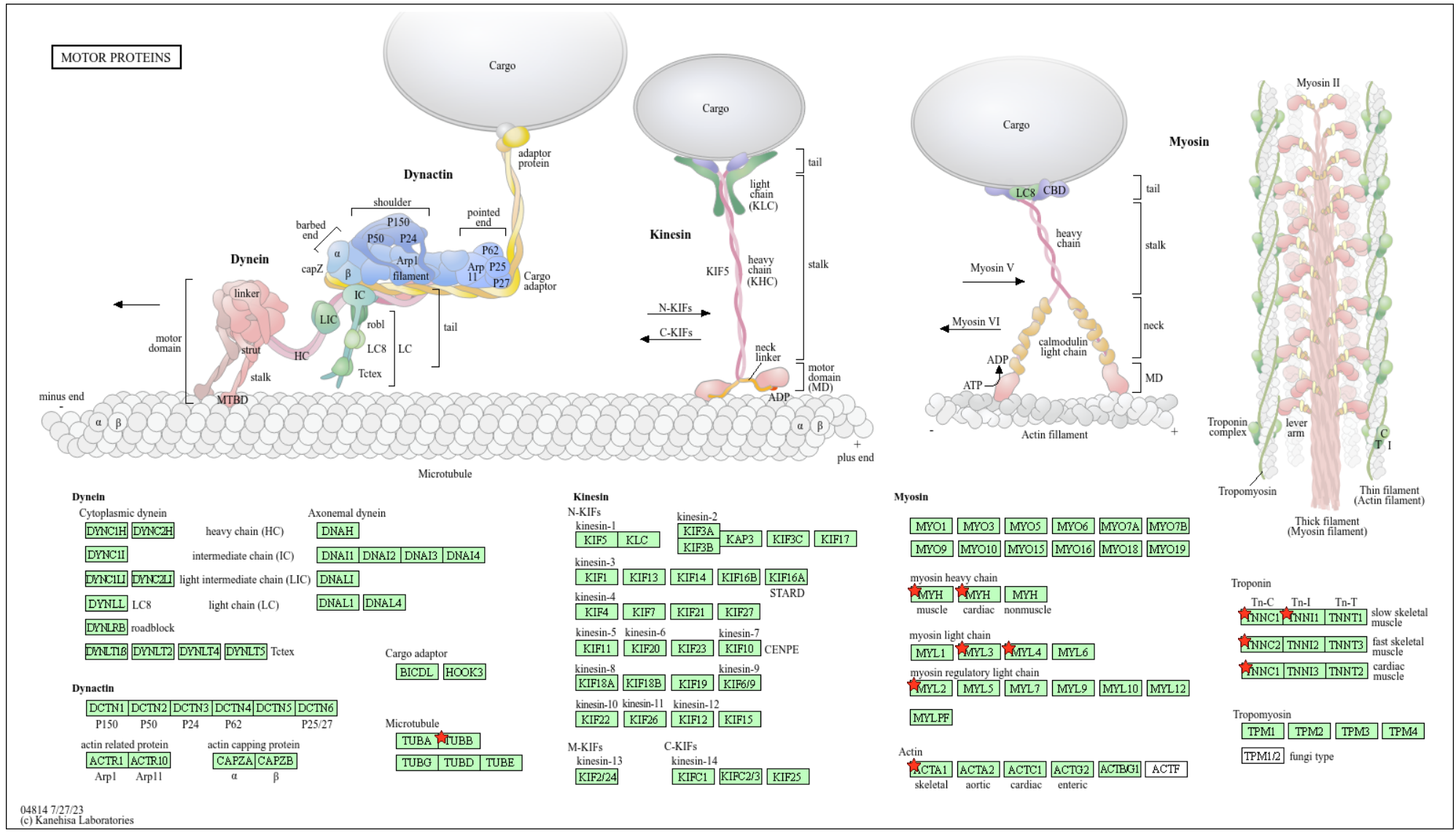

Supplement: Supplementary file 1 — Supplementary file1 (TIF 1198 KB) [file 44164_2025_87_MOESM1_ESM.tif]
