# Supplementary material for: The use of three-dimensional primary human myospheres to explore skeletal muscle effects of in vivo krill oil supplementation
Source: In Vitro Model. 2025 Apr 30;4(2):145–55. doi: 10.1007/s44164-025-00087-6 (PMC12283505; doi:10.1007/s44164-025-00087-6)
Supplement: Supplementary file 2 — Supplementary file2 (DOCX 61 KB) [file 44164_2025_87_MOESM2_ESM.docx]

**The use of three-dimensional primary human myospheres to explore skeletal muscle effects of in vivo krill oil supplementation**

Journal: In vitro models

Andrea Dalmao-Fernandez, Parmeshwar B. Katare, Hege G. Bakke, Håvard Hamarsland, Stian Ellefsen, Sachin Singh, Tuula Anneli Nyman, Eili Tranheim Kase, Arild C. Rustan, G. Hege Thoresen

Section for Pharmacology and Pharmaceutical Biosciences, Department of Pharmacy, University of Oslo, Norway

hege.thoresen@farmasi.uio.no

**Supplementary table 1:** Up and downregulated protein-coding genes in myosphere cultures after *in vivo* krill oil supplementation.

| **Gene ID** | **Gene name** | **Gene description** | **Log2**  **FoldChange** | **-Log10**  **(p-value)** | **Regulated** |
| --- | --- | --- | --- | --- | --- |
| ENSG00000100505 | TRIM9 | tripartite motif containing 9 | 0,60 | 1,60 | Up |
| ENSG00000092969 | TGFB2 | transforming growth factor beta 2 | 0,67 | 1,68 | Up |
| ENSG00000006576 | PHTF2 | putative homeodomain transcription factor 2 | 0,71 | 1,79 | Up |
| ENSG00000168542 | COL3A1 | collagen type III alpha 1 chain | 0,71 | 1,49 | Up |
| ENSG00000148848 | ADAM12 | ADAM metallopeptidase domain 12 | 0,78 | 1,87 | Up |
| ENSG00000188738 | FSIP2 | fibrous sheath interacting protein 2 | 0,83 | 1,53 | Up |
| ENSG00000082512 | TRAF5 | TNF receptor associated factor 5 | 0,84 | 1,50 | Up |
| ENSG00000133315 | MACROD1 | MACRO domain containing 1 | 0,86 | 1,51 | Up |
| ENSG00000011426 | ANLN | anillin actin binding protein | 0,92 | 1,94 | Up |
| ENSG00000185345 | PRKN | parkin RBR E3 ubiquitin protein ligase | 0,94 | 1,45 | Up |
| ENSG00000139985 | ADAM21 | ADAM metallopeptidase domain 21 | 0,96 | 1,68 | Up |
| ENSG00000123080 | CDKN2C | cyclin dependent kinase inhibitor 2C | 1,07 | 1,36 | Up |
| ENSG00000162458 | FBLIM1 | filamin binding LIM protein 1 | 1,07 | 1,42 | Up |
| ENSG00000135048 | CEMIP2 | cell migration inducing hyaluronidase 2 | 1,08 | 1,35 | Up |
| ENSG00000170412 | GPRC5C | G protein-coupled receptor class C: 5 member C | 1,08 | 1,35 | Up |
| ENSG00000101115 | SALL4 | spalt like transcription factor 4 | 1,09 | 1,37 | Up |
| ENSG00000172164 | SNTB1 | syntrophin beta 1 | 1,13 | 1,45 | Up |
| ENSG00000139508 | SLC46A3 | solute carrier family 46 member 3 | 1,15 | 1,33 | Up |
| ENSG00000188687 | SLC4A5 | solute carrier family 4 member 5 | 1,16 | 1,69 | Up |
| ENSG00000182022 | CHST15 | carbohydrate sulfotransferase 15 | 1,17 | 1,38 | Up |
| ENSG00000147145 | LPAR4 | lysophosphatidic acid receptor 4 | 1,18 | 1,59 | Up |
| ENSG00000123095 | BHLHE41 | basic helix-loop-helix family member e41 | 1,20 | 1,73 | Up |
| ENSG00000176842 | IRX5 | iroquois homeobox 5 | 1,21 | 1,69 | Up |
| ENSG00000123096 | SSPN | sarcospan | 1,23 | 1,78 | Up |
| ENSG00000159348 | CYB5R1 | cytochrome b5 reductase 1 | 1,28 | 1,46 | Up |
| ENSG00000135744 | AGT | angiotensinogen | 1,28 | 1,69 | Up |
| ENSG00000106829 | TLE4 | transducin like enhancer of split 4 | 1,28 | 1,52 | Up |
| ENSG00000086570 | FAT2 | FAT atypical cadherin 2 | 1,30 | 1,39 | Up |
| ENSG00000104177 | MYEF2 | myelin expression factor 2 | 1,30 | 1,53 | Up |
| ENSG00000143367 | TUFT1 | tuftelin 1 | 1,30 | 1,59 | Up |
| ENSG00000163618 | CADPS | calcium dependent secretion activator | 1,31 | 1,34 | Up |
| ENSG00000015568 | RGPD5 | RANBP2-like and GRIP domain containing 5 | 1,32 | 1,80 | Up |
| ENSG00000168961 | LGALS9 | galectin 9 | 1,32 | 1,31 | Up |
| ENSG00000089692 | LAG3 | lymphocyte activating 3 | 1,32 | 1,34 | Up |
| ENSG00000151725 | CENPU | centromere protein U | 1,33 | 1,46 | Up |
| ENSG00000262246 | CORO7 | coronin 7 | 1,34 | 1,66 | Up |
| ENSG00000137198 | GMPR | guanosine monophosphate reductase | 1,35 | 1,32 | Up |
| ENSG00000170417 | TMEM182 | transmembrane protein 182 | 1,35 | 1,61 | Up |
| ENSG00000213988 | ZNF90 | zinc finger protein 90 | 1,35 | 1,79 | Up |
| ENSG00000138835 | RGS3 | regulator of G protein signaling 3 | 1,35 | 1,45 | Up |
| ENSG00000137285 | TUBB2B | tubulin beta 2B class IIb | 1,36 | 1,51 | Up |
| ENSG00000148120 | C9orf3 | chromosome 9 open reading frame 3 | 1,36 | 1,51 | Up |
| ENSG00000105088 | OLFM2 | olfactomedin 2 | 1,39 | 1,89 | Up |
| ENSG00000106772 | PRUNE2 | prune homolog 2 | 1,42 | 1,40 | Up |
| ENSG00000106236 | NPTX2 | neuronal pentraxin 2 | 1,43 | 1,41 | Up |
| ENSG00000105808 | RASA4 | RAS p21 protein activator 4 | 1,45 | 2,08 | Up |
| ENSG00000046653 | GPM6B | glycoprotein M6B | 1,45 | 2,29 | Up |
| ENSG00000250486 | FAM218A | family with sequence similarity 218 member A | 1,45 | 1,41 | Up |
| ENSG00000189159 | JPT1 | Jupiter microtubule associated homolog 1 | 1,46 | 1,72 | Up |
| ENSG00000166206 | GABRB3 | gamma-aminobutyric acid type A receptor β3subunit | 1,47 | 1,54 | Up |
| ENSG00000075702 | WDR62 | WD repeat domain 62 | 1,48 | 1,60 | Up |
| ENSG00000070808 | CAMK2A | calcium/calmodulin dependent protein kinase II α | 1,48 | 1,61 | Up |
| ENSG00000122378 | PRXL2A | peroxiredoxin like 2A | 1,49 | 1,72 | Up |
| ENSG00000128487 | SPECC1 | sperm antigen with calponin homology and coiled-coil domains 1 | 1,49 | 1,99 | Up |
| ENSG00000022267 | FHL1 | four and a half LIM domains 1 | 1,49 | 1,84 | Up |
| ENSG00000132613 | MTSS1L | MTSS1L, I-BAR domain containing | 1,49 | 1,37 | Up |
| ENSG00000196497 | IPO4 | importin 4 | 1,50 | 1,37 | Up |
| ENSG00000221955 | SLC12A8 | solute carrier family 12 member 8 | 1,50 | 1,44 | Up |
| ENSG00000143473 | KCNH1 | potassium voltage-gated channel subfamily H member 1 | 1,51 | 1,44 | Up |
| ENSG00000135424 | ITGA7 | integrin subunit alpha 7 | 1,52 | 1,73 | Up |
| ENSG00000152689 | RASGRP3 | RAS guanyl releasing protein 3 | 1,53 | 1,38 | Up |
| ENSG00000104435 | STMN2 | stathmin 2 | 1,54 | 1,78 | Up |
| ENSG00000152422 | XRCC4 | X-ray repair cross complementing 4 | 1,54 | 1,49 | Up |
| ENSG00000135077 | HAVCR2 | hepatitis A virus cellular receptor 2 | 1,54 | 2,60 | Up |
| ENSG00000169242 | EFNA1 | ephrin A1 | 1,55 | 1,86 | Up |
| ENSG00000167900 | TK1 | thymidine kinase 1 | 1,55 | 1,41 | Up |
| ENSG00000138449 | SLC40A1 | solute carrier family 40 member 1 | 1,55 | 1,55 | Up |
| ENSG00000176387 | HSD11B2 | hydroxysteroid 11-beta dehydrogenase 2 | 1,56 | 1,64 | Up |
| ENSG00000254852 | NPIPA2 | nuclear pore complex interacting protein memberA2 | 1,57 | 1,71 | Up |
| ENSG00000116254 | CHD5 | chromodomain helicase DNA binding protein 5 | 1,57 | 1,78 | Up |
| ENSG00000165495 | PKNOX2 | PBX/knotted 1 homeobox 2 | 1,57 | 1,35 | Up |
| ENSG00000149591 | TAGLN | transgelin | 1,60 | 1,50 | Up |
| ENSG00000139269 | INHBE | inhibin subunit beta E | 1,62 | 1,39 | Up |
| ENSG00000001561 | ENPP4 | ectonucleotide pyrophosphatase/phosphodiesterase 4 | 1,62 | 1,38 | Up |
| ENSG00000180914 | OXTR | oxytocin receptor | 1,63 | 1,66 | Up |
| ENSG00000016402 | IL20RA | interleukin 20 receptor subunit alpha | 1,64 | 1,41 | Up |
| ENSG00000172986 | GXYLT2 | glucoside xylosyltransferase 2 | 1,64 | 1,56 | Up |
| ENSG00000148541 | FAM13C | family with sequence similarity 13 member C | 1,65 | 1,99 | Up |
| ENSG00000135324 | MRAP2 | melanocortin 2 receptor accessory protein 2 | 1,67 | 1,61 | Up |
| ENSG00000130222 | GADD45G | growth arrest and DNA damage inducible gamma | 1,68 | 1,43 | Up |
| ENSG00000021645 | NRXN3 | neurexin 3 | 1,68 | 1,33 | Up |
| ENSG00000068976 | PYGM | glycogen phosphorylase, muscle associated | 1,68 | 1,44 | Up |
| ENSG00000170382 | LRRN2 | leucine rich repeat neuronal 2 | 1,70 | 1,52 | Up |
| ENSG00000182580 | EPHB3 | EPH receptor B3 | 1,70 | 1,55 | Up |
| ENSG00000065534 | MYLK | myosin light chain kinase | 1,71 | 1,50 | Up |
| ENSG00000138795 | LEF1 | lymphoid enhancer binding factor 1 | 1,72 | 1,32 | Up |
| ENSG00000172399 | MYOZ2 | myozenin 2 | 1,77 | 1,99 | Up |
| ENSG00000164879 | CA3 | carbonic anhydrase 3 | 1,77 | 1,52 | Up |
| ENSG00000161594 | KLHL10 | kelch like family member 10 | 1,79 | 1,31 | Up |
| ENSG00000240891 | PLCXD2 | phosphatidylinositol specific phospholipase C X domain containing 2 | 1,79 | 1,36 | Up |
| ENSG00000144152 | FBLN7 | fibulin 7 | 1,80 | 1,67 | Up |
| ENSG00000134716 | CYP2J2 | cytochrome P450 family 2 subfamily J member 2 | 1,80 | 1,83 | Up |
| ENSG00000072858 | SIDT1 | SID1 transmembrane family member 1 | 1,83 | 1,48 | Up |
| ENSG00000146005 | PSD2 | pleckstrin and Sec7 domain containing 2 | 1,83 | 1,43 | Up |
| ENSG00000166923 | GREM1 | gremlin 1, DAN family BMP antagonist | 1,84 | 2,02 | Up |
| ENSG00000172346 | CSDC2 | cold shock domain containing C2 | 1,85 | 1,38 | Up |
| ENSG00000170439 | METTL7B | methyltransferase like 7B | 1,86 | 1,69 | Up |
| ENSG00000076864 | RAP1GAP | RAP1 GTPase activating protein | 1,86 | 1,75 | Up |
| ENSG00000136541 | ERMN | ermin | 1,86 | 1,61 | Up |
| ENSG00000101977 | MCF2 | MCF.2 cell line derived transforming sequence | 1,87 | 1,48 | Up |
| ENSG00000111262 | KCNA1 | potassium voltage-gated channel subfamily A member 1 | 1,87 | 1,30 | Up |
| ENSG00000131650 | KREMEN2 | kringle containing transmembrane protein 2 | 1,88 | 1,39 | Up |
| ENSG00000155792 | DEPTOR | DEP domain containing MTOR interacting protein | 1,89 | 1,56 | Up |
| ENSG00000228672 | PROB1 | proline rich basic protein 1 | 1,89 | 1,56 | Up |
| ENSG00000165092 | ALDH1A1 | aldehyde dehydrogenase 1 family member A1 | 1,89 | 1,45 | Up |
| ENSG00000165617 | DACT1 | dishevelled binding antagonist of beta catenin 1 | 1,91 | 1,51 | Up |
| ENSG00000105717 | PBX4 | PBX homeobox 4 | 1,91 | 1,30 | Up |
| ENSG00000099282 | TSPAN15 | tetraspanin 15 | 1,92 | 1,35 | Up |
| ENSG00000126882 | FAM78A | family with sequence similarity 78 member A | 1,94 | 1,79 | Up |
| ENSG00000187244 | BCAM | basal cell adhesion molecule (Lutheran blood group) | 1,95 | 1,84 | Up |
| ENSG00000106483 | SFRP4 | secreted frizzled related protein 4 | 1,97 | 2,29 | Up |
| ENSG00000158528 | PPP1R9A | protein phosphatase 1 regulatory subunit 9A | 1,97 | 1,63 | Up |
| ENSG00000113924 | HGD | homogentisate 1,2-dioxygenase | 1,97 | 1,63 | Up |
| ENSG00000114541 | FRMD4B | FERM domain containing 4B | 1,99 | 1,79 | Up |
| ENSG00000174429 | ABRA | actin binding Rho activating protein | 1,99 | 1,39 | Up |
| ENSG00000108515 | ENO3 | enolase 3 | 1,99 | 2,18 | Up |
| ENSG00000146477 | SLC22A3 | solute carrier family 22 member 3 | 2,01 | 4,10 | Up |
| ENSG00000260027 | HOXB7 | homeobox B7 | 2,02 | 1,64 | Up |
| ENSG00000197616 | MYH6 | myosin heavy chain 6 | 2,02 | 1,65 | Up |
| ENSG00000182010 | RTKN2 | rhotekin 2 | 2,02 | 1,35 | Up |
| ENSG00000197361 | FBXL22 | F-box and leucine rich repeat protein 22 | 2,02 | 1,55 | Up |
| ENSG00000198771 | RCSD1 | RCSD domain containing 1 | 2,04 | 2,04 | Up |
| ENSG00000176383 | B3GNT4 | UDP-GlcNAc:betaGal beta-1,3-N-acetylglucosaminyltransferase 4 | 2,07 | 1,78 | Up |
| ENSG00000215845 | TSTD1 | thiosulfate sulfurtransferase like domain 1 | 2,08 | 1,44 | Up |
| ENSG00000186417 | GLDN | gliomedin | 2,08 | 1,69 | Up |
| ENSG00000172264 | MACROD2 | MACRO domain containing 2 | 2,08 | 1,58 | Up |
| ENSG00000198336 | MYL4 | myosin light chain 4 | 2,08 | 1,33 | Up |
| ENSG00000224940 | PRRT4 | proline rich transmembrane protein 4 | 2,09 | 1,50 | Up |
| ENSG00000103742 | IGDCC4 | immunoglobulin superfamily DCC subclass 4 | 2,09 | 1,60 | Up |
| ENSG00000127528 | KLF2 | Kruppel like factor 2 | 2,14 | 1,76 | Up |
| ENSG00000101825 | MXRA5 | matrix remodeling associated 5 | 2,15 | 1,36 | Up |
| ENSG00000177752 | YIPF7 | Yip1 domain family member 7 | 2,15 | 1,80 | Up |
| ENSG00000142621 | FHAD1 | forkhead associated phosphopeptide binding 1 | 2,15 | 3,14 | Up |
| ENSG00000187800 | PEAR1 | platelet endothelial aggregation receptor 1 | 2,15 | 1,88 | Up |
| ENSG00000164440 | TXLNB | taxilin beta | 2,17 | 2,07 | Up |
| ENSG00000165449 | SLC16A9 | solute carrier family 16 member 9 | 2,18 | 2,18 | Up |
| ENSG00000167588 | GPD1 | glycerol-3-phosphate dehydrogenase 1 | 2,18 | 1,42 | Up |
| ENSG00000189350 | TOGARAM2 | TOG array regulator of axonemal microtubules 2 | 2,18 | 1,88 | Up |
| ENSG00000196296 | ATP2A1 | ATPase sarcoplasmic/endoplasmic reticulum Ca2+ transporting 1 | 2,18 | 2,29 | Up |
| ENSG00000232237 | ASCL5 | achaete-scute family bHLH transcription factor 5 | 2,19 | 1,79 | Up |
| ENSG00000163995 | ABLIM2 | actin binding LIM protein family member 2 | 2,25 | 1,34 | Up |
| ENSG00000064787 | BCAS1 | breast carcinoma amplified sequence 1 | 2,26 | 3,68 | Up |
| ENSG00000108602 | ALDH3A1 | aldehyde dehydrogenase 3 family member A1 | 2,26 | 1,37 | Up |
| ENSG00000162391 | FAM151A | family with sequence similarity 151 member A | 2,27 | 1,41 | Up |
| ENSG00000108639 | SYNGR2 | synaptogyrin 2 | 2,28 | 1,71 | Up |
| ENSG00000173930 | SLCO4C1 | solute carrier organic anion transporter family member 4C1 | 2,29 | 1,60 | Up |
| ENSG00000122477 | LRRC39 | leucine rich repeat containing 39 | 2,29 | 2,43 | Up |
| ENSG00000170891 | CYTL1 | cytokine like 1 | 2,34 | 1,88 | Up |
| ENSG00000175920 | DOK7 | docking protein 7 | 2,35 | 1,38 | Up |
| ENSG00000157851 | DPYSL5 | dihydropyrimidinase like 5 | 2,35 | 2,01 | Up |
| ENSG00000109943 | CRTAM | cytotoxic and regulatory T cell molecule | 2,36 | 1,67 | Up |
| ENSG00000212993 | POU5F1B | POU class 5 homeobox 1B | 2,37 | 1,88 | Up |
| ENSG00000164684 | ZNF704 | zinc finger protein 704 | 2,38 | 2,17 | Up |
| ENSG00000138615 | CILP | cartilage intermediate layer protein | 2,38 | 1,80 | Up |
| ENSG00000114115 | RBP1 | retinol binding protein 1 | 2,38 | 1,63 | Up |
| ENSG00000142583 | SLC2A5 | solute carrier family 2 member 5 | 2,38 | 1,89 | Up |
| ENSG00000040608 | RTN4R | reticulon 4 receptor | 2,40 | 1,53 | Up |
| ENSG00000151715 | TMEM45B | transmembrane protein 45B | 2,41 | 1,70 | Up |
| ENSG00000184368 | MAP7D2 | MAP7 domain containing 2 | 2,41 | 2,61 | Up |
| ENSG00000125414 | MYH2 | myosin heavy chain 2 | 2,42 | 1,58 | Up |
| ENSG00000091482 | SMPX | small muscle protein X-linked | 2,43 | 1,51 | Up |
| ENSG00000133317 | LGALS12 | galectin 12 | 2,43 | 1,38 | Up |
| ENSG00000213892 | CEACAM16 | carcinoembryonic antigen related cell adhesion molecule 16 | 2,44 | 1,73 | Up |
| ENSG00000188338 | SLC38A3 | solute carrier family 38 member 3 | 2,44 | 1,94 | Up |
| ENSG00000169271 | HSPB3 | heat shock protein family B (small) member 3 | 2,45 | 1,53 | Up |
| ENSG00000101210 | EEF1A2 | eukaryotic translation elongation factor 1 alpha 2 | 2,48 | 2,52 | Up |
| ENSG00000168754 | FAM178B | family with sequence similarity 178 member B | 2,48 | 1,43 | Up |
| ENSG00000203499 | IQANK1 | IQ motif and ankyrin repeat containing 1 | 2,50 | 2,31 | Up |
| ENSG00000133985 | TTC9 | tetratricopeptide repeat domain 9 | 2,52 | 1,91 | Up |
| ENSG00000121966 | CXCR4 | C-X-C motif chemokine receptor 4 | 2,53 | 1,51 | Up |
| ENSG00000114854 | TNNC1 | troponin C1, slow skeletal and cardiac type | 2,55 | 1,92 | Up |
| ENSG00000243978 | RTL9 | retrotransposon Gag like 9 | 2,56 | 1,40 | Up |
| ENSG00000147166 | ITGB1BP2 | integrin subunit beta 1 binding protein 2 | 2,56 | 2,12 | Up |
| ENSG00000184113 | CLDN5 | claudin 5 | 2,57 | 1,46 | Up |
| ENSG00000003249 | DBNDD1 | dysbindin domain containing 1 | 2,58 | 2,20 | Up |
| ENSG00000151468 | CCDC3 | coiled-coil domain containing 3 | 2,59 | 1,91 | Up |
| ENSG00000122180 | MYOG | myogenin | 2,59 | 1,68 | Up |
| ENSG00000169129 | AFAP1L2 | actin filament associated protein 1 like 2 | 2,60 | 1,84 | Up |
| ENSG00000036448 | MYOM2 | myomesin 2 | 2,60 | 2,17 | Up |
| ENSG00000181418 | DDN | dendrin | 2,60 | 2,12 | Up |
| ENSG00000076554 | TPD52 | tumor protein D52 | 2,61 | 1,81 | Up |
| ENSG00000239474 | KLHL41 | kelch like family member 41 | 2,62 | 2,01 | Up |
| ENSG00000182676 | PPP1R27 | protein phosphatase 1 regulatory subunit 27 | 2,63 | 4,59 | Up |
| ENSG00000036672 | USP2 | ubiquitin specific peptidase 2 | 2,64 | 2,25 | Up |
| ENSG00000133055 | MYBPH | myosin binding protein H | 2,64 | 1,84 | Up |
| ENSG00000171004 | HS6ST2 | heparan sulfate 6-O-sulfotransferase 2 | 2,64 | 1,82 | Up |
| ENSG00000174600 | CMKLR1 | chemerin chemokine-like receptor 1 | 2,65 | 3,01 | Up |
| ENSG00000159261 | CLDN14 | claudin 14 | 2,67 | 2,02 | Up |
| ENSG00000159173 | TNNI1 | troponin I1, slow skeletal type | 2,70 | 1,87 | Up |
| ENSG00000156298 | TSPAN7 | tetraspanin 7 | 2,74 | 2,65 | Up |
| ENSG00000010319 | SEMA3G | semaphorin 3G | 2,74 | 1,60 | Up |
| ENSG00000158457 | TSPAN33 | tetraspanin 33 | 2,75 | 3,19 | Up |
| ENSG00000006788 | MYH13 | myosin heavy chain 13 | 2,82 | 1,50 | Up |
| ENSG00000111046 | MYF6 | myogenic factor 6 | 2,84 | 1,61 | Up |
| ENSG00000173991 | TCAP | titin-cap | 2,86 | 3,03 | Up |
| ENSG00000183230 | CTNNA3 | catenin alpha 3 | 2,86 | 2,47 | Up |
| ENSG00000106714 | CNTNAP3 | contactin associated protein like 3 | 2,88 | 1,32 | Up |
| ENSG00000155629 | PIK3AP1 | phosphoinositide-3-kinase adaptor protein 1 | 2,88 | 1,95 | Up |
| ENSG00000198523 | PLN | phospholamban | 2,91 | 2,58 | Up |
| ENSG00000159516 | SPRR2G | small proline rich protein 2G | 2,91 | 1,52 | Up |
| ENSG00000010327 | STAB1 | stabilin 1 | 2,92 | 1,89 | Up |
| ENSG00000158022 | TRIM63 | tripartite motif containing 63 | 2,93 | 1,68 | Up |
| ENSG00000110195 | FOLR1 | folate receptor 1 | 2,94 | 2,02 | Up |
| ENSG00000257017 | HP | haptoglobin | 2,94 | 2,22 | Up |
| ENSG00000070031 | SCT | secretin | 2,95 | 2,44 | Up |
| ENSG00000123329 | ARHGAP9 | Rho GTPase activating protein 9 | 3,02 | 2,21 | Up |
| ENSG00000113296 | THBS4 | thrombospondin 4 | 3,04 | 2,61 | Up |
| ENSG00000010282 | HHATL | hedgehog acyltransferase like | 3,06 | 1,77 | Up |
| ENSG00000137801 | THBS1 | thrombospondin 1 | 3,06 | 2,93 | Up |
| ENSG00000133878 | DUSP26 | dual specificity phosphatase 26 | 3,07 | 2,26 | Up |
| ENSG00000180999 | C1orf105 | chromosome 1 open reading frame 105 | 3,11 | 2,07 | Up |
| ENSG00000170927 | PKHD1 | PKHD1, fibrocystin/polyductin | 3,16 | 1,64 | Up |
| ENSG00000101470 | TNNC2 | troponin C2, fast skeletal type | 3,18 | 2,85 | Up |
| ENSG00000155511 | GRIA1 | glutamate ionotropic receptor AMPA type subunit 1 | 3,18 | 2,80 | Up |
| ENSG00000100604 | CHGA | chromogranin A | 3,19 | 1,37 | Up |
| ENSG00000111245 | MYL2 | myosin light chain 2 | 3,23 | 3,37 | Up |
| ENSG00000154415 | PPP1R3A | protein phosphatase 1 regulatory subunit 3A | 3,23 | 1,78 | Up |
| ENSG00000160808 | MYL3 | myosin light chain 3 | 3,25 | 2,81 | Up |
| ENSG00000092054 | MYH7 | myosin heavy chain 7 | 3,28 | 2,69 | Up |
| ENSG00000099958 | DERL3 | derlin 3 | 3,29 | 1,45 | Up |
| ENSG00000187862 | TTC24 | tetratricopeptide repeat domain 24 | 3,32 | 1,39 | Up |
| ENSG00000186628 | FSD2 | fibronectin type III and SPRY domain containing 2 | 3,35 | 1,87 | Up |
| ENSG00000267060 | PTGES3L | prostaglandin E synthase 3 like | 3,35 | 2,42 | Up |
| ENSG00000104537 | ANXA13 | annexin A13 | 3,42 | 1,82 | Up |
| ENSG00000165325 | DEUP1 | deuterosome assembly protein 1 | 3,46 | 1,57 | Up |
| ENSG00000120729 | MYOT | myotilin | 3,48 | 3,60 | Up |
| ENSG00000106078 | COBL | cordon-bleu WH2 repeat protein | 3,48 | 2,55 | Up |
| ENSG00000214097 | SMCO1 | single-pass membrane protein with coiled-coil domains 1 | 3,55 | 1,32 | Up |
| ENSG00000143632 | ACTA1 | actin, alpha 1, skeletal muscle | 3,63 | 3,05 | Up |
| ENSG00000153930 | ANKFN1 | ankyrin repeat and fibronectin type III domain containing 1 | 3,64 | 1,72 | Up |
| ENSG00000117650 | NEK2 | NIMA related kinase 2 | 3,67 | 1,39 | Up |
| ENSG00000187715 | KBTBD12 | kelch repeat and BTB domain containing 12 | 3,74 | 1,81 | Up |
| ENSG00000180432 | CYP8B1 | cytochrome P450 family 8 subfamily B member 1 | 3,81 | 1,82 | Up |
| ENSG00000120332 | TNN | tenascin N | 3,87 | 1,84 | Up |
| ENSG00000185640 | KRT79 | keratin 79 | 3,87 | 1,62 | Up |
| ENSG00000178562 | CD28 | CD28 molecule | 3,92 | 1,64 | Up |
| ENSG00000132321 | IQCA1 | IQ motif containing with AAA domain 1 | 3,96 | 1,75 | Up |
| ENSG00000160862 | AZGP1 | alpha-2-glycoprotein 1, zinc-binding | 3,96 | 1,43 | Up |
| ENSG00000205426 | KRT81 | keratin 81 | 4,00 | 1,67 | Up |
| ENSG00000100628 | ASB2 | ankyrin repeat and SOCS box containing 2 | 4,01 | 2,37 | Up |
| ENSG00000170549 | IRX1 | iroquois homeobox 1 | 4,01 | 1,57 | Up |
| ENSG00000171714 | ANO5 | anoctamin 5 | 4,04 | 3,75 | Up |
| ENSG00000102678 | FGF9 | fibroblast growth factor 9 | 4,10 | 1,45 | Up |
| ENSG00000188910 | GJB3 | gap junction protein beta 3 | 4,16 | 1,65 | Up |
| ENSG00000170091 | NSG2 | neuronal vesicle trafficking associated 2 | 4,18 | 1,54 | Up |
| ENSG00000175946 | KLHL38 | kelch like family member 38 | 4,20 | 1,35 | Up |
| ENSG00000197893 | NRAP | nebulin related anchoring protein | 4,21 | 4,24 | Up |
| ENSG00000104879 | CKM | creatine kinase, M-type | 4,22 | 3,73 | Up |
| ENSG00000099985 | OSM | oncostatin M | 4,28 | 1,33 | Up |
| ENSG00000129170 | CSRP3 | cysteine and glycine rich protein 3 | 4,31 | 2,21 | Up |
| ENSG00000264424 | MYH4 | myosin heavy chain 4 | 4,34 | 2,04 | Up |
| ENSG00000197406 | DIO3 | iodothyronine deiodinase 3 | 4,35 | 1,90 | Up |
| ENSG00000248713 | C4orf54 | chromosome 4 open reading frame 54 | 4,38 | 3,29 | Up |
| ENSG00000075461 | CACNG4 | calcium voltage-gated channel auxiliary subunit gamma 4 | 4,42 | 1,69 | Up |
| ENSG00000169627 | BOLA2B | bolA family member 2B | 4,49 | 3,98 | Up |
| ENSG00000129744 | ART1 | ADP-ribosyltransferase 1 | 4,52 | 1,90 | Up |
| ENSG00000134323 | MYCN | MYCN proto-oncogene, bHLH transcription factor | 4,66 | 2,14 | Up |
| ENSG00000184811 | TRARG1 | trafficking regulator of GLUT4 (SLC2A4) 1 | 4,69 | 1,50 | Up |
| ENSG00000163092 | XIRP2 | xin actin binding repeat containing 2 | 4,91 | 4,53 | Up |
| ENSG00000101306 | MYLK2 | myosin light chain kinase 2 | 5,11 | 1,94 | Up |
| ENSG00000138823 | MTTP | microsomal triglyceride transfer protein | 5,14 | 3,03 | Up |
| ENSG00000116748 | AMPD1 | adenosine monophosphate deaminase 1 | 5,22 | 2,31 | Up |
| ENSG00000115263 | GCG | glucagon | 5,22 | 1,42 | Up |
| ENSG00000162399 | BSND | barttin CLCNK type accessory beta subunit | 5,33 | 1,46 | Up |
| ENSG00000163833 | FBXO40 | F-box protein 40 | 5,43 | 1,75 | Up |
| ENSG00000177354 | C10orf71 | chromosome 10 open reading frame 71 | 5,49 | 4,52 | Up |
| ENSG00000177791 | MYOZ1 | myozenin 1 | 6,58 | 4,01 | Up |
| ENSG00000171509 | RXFP1 | relaxin family peptide receptor 1 | -4,57 | 4,65 | Down |
| ENSG00000196242 | OR2C3 | olfactory receptor family 2 subfamily C member 3 | -4,31 | 1,46 | Down |
| ENSG00000189233 | NUGGC | nuclear GTPase, germinal center associated | -3,65 | 1,77 | Down |
| ENSG00000178233 | TMEM151B | transmembrane protein 151B | -3,64 | 1,80 | Down |
| ENSG00000156687 | UNC5D | unc-5 netrin receptor D | -3,47 | 1,92 | Down |
| ENSG00000203837 | PNLIPRP3 | pancreatic lipase related protein 3 | -3,46 | 2,55 | Down |
| ENSG00000149968 | MMP3 | matrix metallopeptidase 3 | -3,29 | 1,34 | Down |
| ENSG00000167612 | ANKRD33 | ankyrin repeat domain 33 | -3,25 | 2,14 | Down |
| ENSG00000186118 | TEX38 | testis expressed 38 | -3,17 | 1,36 | Down |
| ENSG00000204252 | HLA-DOA | major histocompatibility complex, class II, DO α | -2,88 | 1,50 | Down |
| ENSG00000204287 | HLA-DRA | major histocompatibility complex, class II, DR α | -2,67 | 1,98 | Down |
| ENSG00000136944 | LMX1B | LIM homeobox transcription factor 1 beta | -2,66 | 1,52 | Down |
| ENSG00000103154 | NECAB2 | N-terminal EF-hand calcium binding protein 2 | -2,58 | 2,09 | Down |
| ENSG00000108622 | ICAM2 | intercellular adhesion molecule 2 | -2,54 | 3,07 | Down |
| ENSG00000172543 | CTSW | cathepsin W | -2,49 | 1,70 | Down |
| ENSG00000156414 | TDRD9 | tudor domain containing 9 | -2,36 | 1,46 | Down |
| ENSG00000205609 | EIF3CL | eukaryotic translation initiation factor 3 subunC like | -2,36 | 2,38 | Down |
| ENSG00000179583 | CIITA | class II major histocompatibility complex transactivator | -2,35 | 2,51 | Down |
| ENSG00000181634 | TNFSF15 | TNF superfamily member 15 | -2,25 | 1,35 | Down |
| ENSG00000205835 | GMNC | geminin coiled-coil domain containing | -2,11 | 2,11 | Down |
| ENSG00000217236 | SP9 | Sp9 transcription factor | -2,10 | 1,32 | Down |
| ENSG00000119547 | ONECUT2 | one cut homeobox 2 | -2,09 | 1,79 | Down |
| ENSG00000168878 | SFTPB | surfactant protein B | -1,97 | 1,36 | Down |
| ENSG00000187758 | ADH1A | alcohol dehydrogenase 1A (class I), α polypeptide | -1,95 | 1,34 | Down |
| ENSG00000125735 | TNFSF14 | TNF superfamily member 14 | -1,92 | 1,77 | Down |
| ENSG00000163564 | PYHIN1 | pyrin and HIN domain family member 1 | -1,83 | 1,40 | Down |
| ENSG00000128886 | ELL3 | elongation factor for RNA polymerase II 3 | -1,82 | 1,39 | Down |
| ENSG00000109272 | PF4V1 | platelet factor 4 variant 1 | -1,81 | 1,84 | Down |
| ENSG00000172927 | MYEOV | myeloma overexpressed | -1,77 | 1,94 | Down |
| ENSG00000100558 | PLEK2 | pleckstrin 2 | -1,67 | 2,67 | Down |
| ENSG00000168621 | GDNF | glial cell derived neurotrophic factor | -1,65 | 1,79 | Down |
| ENSG00000198113 | TOR4A | torsin family 4 member A | -1,60 | 1,54 | Down |
| ENSG00000142677 | IL22RA1 | interleukin 22 receptor subunit alpha 1 | -1,57 | 1,76 | Down |
| ENSG00000135114 | OASL | 2'-5'-oligoadenylate synthetase like | -1,51 | 2,26 | Down |
| ENSG00000156463 | SH3RF2 | SH3 domain containing ring finger 2 | -1,51 | 3,02 | Down |
| ENSG00000090554 | FLT3LG | fms related tyrosine kinase 3 ligand | -1,48 | 1,72 | Down |
| ENSG00000160886 | LY6K | lymphocyte antigen 6 family member K | -1,46 | 1,40 | Down |
| ENSG00000144821 | MYH15 | myosin heavy chain 15 | -1,45 | 1,58 | Down |
| ENSG00000197915 | HRNR | hornerin | -1,44 | 1,89 | Down |
| ENSG00000253537 | PCDHGA7 | protocadherin gamma subfamily A, 7 | -1,44 | 3,46 | Down |
| ENSG00000003400 | CASP10 | caspase 10 | -1,43 | 1,65 | Down |
| ENSG00000104321 | TRPA1 | transient receptor potential cation channel subfamily A member 1 | -1,33 | 1,37 | Down |
| ENSG00000010610 | CD4 | CD4 molecule | -1,30 | 1,33 | Down |
| ENSG00000139629 | GALNT6 | polypeptide N-acetylgalactosaminyltransferase 6 | -1,29 | 1,31 | Down |
| ENSG00000164171 | ITGA2 | integrin subunit alpha 2 | -1,25 | 1,59 | Down |
| ENSG00000130988 | RGN | regucalcin | -1,24 | 1,38 | Down |
| ENSG00000198959 | TGM2 | transglutaminase 2 | -1,23 | 1,57 | Down |
| ENSG00000148082 | SHC3 | SHC adaptor protein 3 | -1,21 | 1,33 | Down |
| ENSG00000156218 | ADAMTSL3 | ADAMTS like 3 | -1,12 | 1,32 | Down |
| ENSG00000262209 | PCDHGB3 | protocadherin gamma subfamily B, 3 | -1,09 | 1,87 | Down |
| ENSG00000143674 | MAP3K21 | mitogen-activated protein kinase kinase kinase 21 | -1,06 | 2,15 | Down |
| ENSG00000161642 | ZNF385A | zinc finger protein 385A | -1,04 | 1,40 | Down |
| ENSG00000119922 | IFIT2 | interferon induced protein with tetratricopeptide repeats 2 | -1,01 | 1,64 | Down |
| ENSG00000096433 | ITPR3 | inositol 1,4,5-trisphosphate receptor type 3 | -0,93 | 2,35 | Down |
| ENSG00000140853 | NLRC5 | NLR family CARD domain containing 5 | -0,92 | 1,75 | Down |
| ENSG00000010030 | ETV7 | ETS variant 7 | -0,88 | 2,00 | Down |
| ENSG00000119917 | IFIT3 | interferon induced protein with tetratricopeptide repeats 3 | -0,83 | 2,25 | Down |
| ENSG00000187266 | EPOR | erythropoietin receptor | -0,78 | 1,63 | Down |
| ENSG00000159733 | ZFYVE28 | zinc finger FYVE-type containing 28 | -0,74 | 1,44 | Down |
| ENSG00000130589 | HELZ2 | helicase with zinc finger 2 | -0,74 | 1,30 | Down |
| ENSG00000110090 | CPT1A | carnitine palmitoyltransferase 1A | -0,74 | 1,66 | Down |
| ENSG00000117298 | ECE1 | endothelin converting enzyme 1 | -0,74 | 1,45 | Down |
| ENSG00000253731 | PCDHGA6 | protocadherin gamma subfamily A, 6 | -0,73 | 1,34 | Down |
| ENSG00000185112 | FAM43A | family with sequence similarity 43 member A | -0,72 | 1,86 | Down |
| ENSG00000262576 | PCDHGA4 | protocadherin gamma subfamily A, 4 | -0,69 | 1,32 | Down |
| ENSG00000119714 | GPR68 | G protein-coupled receptor 68 | -0,63 | 2,32 | Down |
| ENSG00000069020 | MAST4 | microtubule associated serine/threonine kinase family member 4 | -0,56 | 2,53 | Down |
| ENSG00000115840 | SLC25A12 | solute carrier family 25 member 12 | -0,40 | 1,38 | Down |
