# Supplementary material for: The use of three-dimensional primary human myospheres to explore skeletal muscle effects of in vivo krill oil supplementation
Source: In Vitro Model. 2025 Apr 30;4(2):145–55. doi: 10.1007/s44164-025-00087-6 (PMC12283505; doi:10.1007/s44164-025-00087-6)
Supplement: Supplementary file 4 — Supplementary file4 (DOCX 16 KB) [file 44164_2025_87_MOESM4_ESM.docx]

**The use of three-dimensional primary human myospheres to explore skeletal muscle effects of in vivo krill oil supplementation**

Journal: In vitro models

Andrea Dalmao-Fernandez, Parmeshwar B. Katare, Hege G. Bakke, Håvard Hamarsland, Stian Ellefsen, Sachin Singh, Tuula Anneli Nyman, Eili Tranheim Kase, Arild C. Rustan, G. Hege Thoresen

Section for Pharmacology and Pharmaceutical Biosciences, Department of Pharmacy, University of Oslo, Norway

[hege.thoresen@farmasi.uio.no](mailto:hege.thoresen@farmasi.uio.no)

**Supplementary table 2:**

**Common reglulated protein-coding genes in 2D and 3D myotube cultures after *in vivo* krill oil supplementation**

| **Gene ID** | **Gene name** | **Gene description** | **Regulated** |
| --- | --- | --- | --- |
| ENSG00000153930 | ANKFN1 | ankyrin repeat and fibronectin type III domain containing 1 | Up |
| ENSG00000196296 | ATP2A1 | ATPase sarcoplasmic/endoplasmic reticulum Ca2+ transporting 1 | Up |
| ENSG00000135048 | CEMIP2 | cell migration inducing hyaluronidase 2 | Up |
| ENSG00000182022 | CHST15 | carbohydrate sulfotransferase 15 | Up |
| ENSG00000174600 | CMKLR1 | chemerin chemokine-like receptor 1 | Up |
| ENSG00000181418 | DDN | dendrin | Up |
| ENSG00000197406 | DIO3 | iodothyronine deiodinase 3 | Up |
| ENSG00000102678 | FGF9 | fibroblast growth factor 9 | Up |
| ENSG00000142621 | FHAD1 | forkhead associated phosphopeptide binding domain 1 | Up |
| ENSG00000184368 | MAP7D2 | MAP7 domain containing 2 | Up |
| ENSG00000006788 | MYH13 | myosin heavy chain 13 | Up |
| ENSG00000106236 | NPTX2 | neuronal pentraxin 2 | Up |
| ENSG00000182676 | PPP1R27 | protein phosphatase 1 regulatory subunit 27 | Up |
| ENSG00000228672 | PROB1 | proline rich basic protein 1 | Up |
| ENSG00000040608 | RTN4R | reticulon 4 receptor | Up |
| ENSG00000221955 | SLC12A8 | solute carrier family 12 member 8 | Up |
| ENSG00000146477 | SLC22A3 | solute carrier family 22 member 3 | Up |
| ENSG00000188338 | SLC38A3 | solute carrier family 38 member 3 | Up |
| ENSG00000137801 | THBS1 | thrombospondin 1 | Up |
| ENSG00000197915 | HRNR | hornerin | Down |
